# Non-Redundant Selector and Growth-Promoting Functions of Two Sister Genes, *buttonhead* and *Sp1*, in *Drosophila* Leg Development

**DOI:** 10.1371/journal.pgen.1001001

**Published:** 2010-06-24

**Authors:** Carlos Estella, Richard S. Mann

**Affiliations:** Department of Biochemistry and Molecular Biophysics, Columbia University, New York, New York, United States of America; Princeton University, Howard Hughes Medical Institute, United States of America

## Abstract

The radically distinct morphologies of arthropod and tetrapod legs argue that these appendages do not share a common evolutionary origin. Yet, despite dramatic differences in morphology, it has been known for some time that transcription factors encoded by the *Distalless* (*Dll*)/*Dlx* gene family play a critical role in the development of both structures. Here we show that a second transcription factor family encoded by the *Sp8* gene family, previously implicated in vertebrate limb development, also plays an early and fundamental role in arthropod leg development. By simultaneously removing the function of two *Sp8* orthologs, *buttonhead* (*btd*) and *Sp1*, during *Drosophila* embryogenesis, we find that adult leg development is completely abolished. Remarkably, in the absence of these factors, transformations from ventral to dorsal appendage identities are observed, suggesting that adult dorsal fates become derepressed when ventral fates are eliminated. Further, we show that *Sp1* plays a much more important role in ventral appendage specification than *btd* and that *Sp1* lies genetically upstream of *Dll.* In addition to these selector-like gene functions, *Sp1* and *btd* are also required during larval stages for the growth of the leg. Vertebrate *Sp8* can rescue many of the functions of the *Drosophila* genes, arguing that these activities have been conserved, despite more than 500 million years of independent evolution. These observations suggest that an ancient *Sp8/Dlx* gene cassette was used in an early metazoan for primitive limb-like outgrowths and that this cassette was co-opted multiple times for appendage formation in multiple animal phyla.

## Author Summary

The development of vertebrate and invertebrate appendages differs in many respects. Yet, despite these differences, genes related to the *Distalless* (*Dll*) gene of *Drosophila* (vertebrate *Dlx* genes) are important for the development of appendages in multiple animal phyla. Such findings raise the question of whether disparate animal appendages have a common evolutionary origin. In vertebrates, a second gene family, related to *Drosophila Sp1*, also plays a fundamental role in appendage development. Although there was some evidence to suggest that *Sp1* family members may play a role in *Drosophila* appendage development, definitive data were lacking. Using a new deficiency that removes both *Drosophila* sister genes, *Sp1* and *buttonhead* (*btd*), we unambiguously assess their role in *Drosophila* development. We find that *Sp1*, but not *btd*, is critical for specifying leg (ventral) development, and that neither gene is required for wing (dorsal) development. We also show that *Sp1* lies genetically upstream of *Dll*. The fact that both *Sp1* and *Dlx* gene families are used for appendage development in vertebrates and invertebrates provides striking evidence that the *Sp1–Dlx* relationship represents an ancient gene network that was used in a common ancestor for appendage-like outgrowths.

## Introduction

During *Drosophila* embryogenesis, the cells that will give rise to both the dorsal (wing and haltere) and ventral (leg) appendages are allocated from a ventral region of each thoracic hemisegment [1], [2]. About a quarter of the way through embryogenesis (stage 11), these cells can be recognized by the expression of the homeobox gene *Distalless* (*Dll*) [3]. Initially, ∼30 ventral cells activate *Dll* in response to receiving positive input from Wingless (Wg) and negative inputs from the Decapentaplegic (Dpp) and Epidermal Growth Factor (EGF) pathways [1], [4],[5]. At this early stage, these *Dll*-expressing cells can contribute to any part of the leg or to any part of the wing and haltere; the distinction between ventral and dorsal appendage identities has not yet occurred [2], [6]. A few hours later (by stage 14), the cells that will give rise to the wing and haltere no longer express *Dll*, and the *Dll*-expressing cells will only contribute to the portion of the leg that is distal to the coxa, the telopodite [2], [7]. Thus, within a few hours, the *Dll*-expressing cells in the thorax have dramatically changed their presumptive fates. This refinement of developmental potential mirrors a change in the *cis-*regulatory elements used to control *Dll* expression. At stage 11, when the *Dll*-expressing cells are multi-potent, *Dll* is activated by the *Dll-304* enhancer [2], [8]. At stage 14, when the fate of *Dll*-expressing cells is limited to the telopodite, *Dll-304* is no longer active and a different regulatory element, *Dll-LT*, is used [2].

Loss-of-function experiments demonstrate that in the absence of *Dll* the leg telopodite fails to develop [1], [9]. Conversely, mis-expression of *Dll* in dorsal appendages can lead to the ectopic development of distal leg segments, most typically, tarsal segments [10]. The necessity and in some contexts sufficiency of *Dll* to generate leg fates has been interpreted to suggest that *Dll* is a selector gene for the ventral appendage. However, although *Dll* is transiently expressed in cells that will give rise to the coxa and dorsal appendages, it is not required to generate these fates, nor is it required to generate any other ventral structure besides the telopodite [1], [9], [10]. Most strikingly, when transplanted to wild type hosts, ventral tissue dissected from *Dll* null embryos retains the capacity to generate proximal leg fates, demonstrating that ventral appendage specification and, in particular, proximal leg fates, form independently of *Dll* [1]. The limited requirement of *Dll* in leg development raises the question of what gene(s) may be required to initially specify the ventral appendage primordia and proximal leg fates.

A pair of genes that could fulfill a more general role in ventral appendage specification is *buttonhead* (*btd*) and *Sp1*, which encode highly related transcription factors with three C2H2 Zn-fingers and a conserved ‘Btd box’ [11], [12]. Both genes share a similar expression pattern throughout *Drosophila* development and appear to have partially redundant functions during mechanosensory organ development [13], [14]. *btd* and *Sp1* are also both expressed in the thoracic appendage primordia (see Figure S1) [14]. Although previous work in *Drosophila* suggested that one or both of these genes may play a role in ventral appendage specification, these conclusions have significant limitations [14]. Embryos homozygous for a large deficiency that removes both *btd* and *Sp1* do not express *Dll* in the ventral primordia [14]. In fact, these embryos appear to have no ventral appendage primordia because expression of *escargot* (*esg*), a general marker for imaginal disc fates, is absent in the ventral thoracic segments of these embryos [14]. However, the large size of the deficiency used in these experiments (*Df(1)C52*), which removes >50 genes in addition to *btd* and *Sp1*, leaves open the question of whether these phenotypes are due to the loss of *btd*, *Sp1*, and/or one of the other deleted genes. Second, mis-expression experiments suggest that Btd has the ability to induce ectopic leg development and the expression of leg marker genes in dorsal tissues, such as the wing. *Sp1*'s activity was not tested in this ectopic expression test [14]. Third, because *Df(1)C52* is too large to be used for clonal analysis, loss-of-function *btd* and *Sp1* phenotypes in the adult were analyzed by RNA interference (RNAi) [14]. Counter to the idea that these genes are essential for leg specification, RNAi knockdown of *btd* and *Sp1* did not prevent leg development, but instead only resulted in a reduction of leg growth. These phenotypes are highly reminiscent to those observed when *btd* and *Sp1* orthologs were knocked-down in the beetle *Tribolium castaneum* (*Sp8*) or milkweed bug *Oncopeltus fasciatus* (*Sp8* and *Sp9*) [15], [16]. In sum, because previous experiments depended on a large deficiency, ectopic expression, and RNAi knockdown approaches, they do not resolve whether *btd* and/or *Sp1* are required for the initial establishment of the ventral appendage primordia and/or for ventral appendage growth.

In vertebrates, there are two genes that are closely related to *btd* and *Sp1*, called *Sp8* and *Sp9*. Both *Sp8* and *Sp9* are initially expressed in the ectoderm of the developing limb bud but are later restricted to the Apical Ectodermal Ridge (AER) [17], [18]. Interestingly, *Sp8* mutant mice have truncated limbs, due to the loss of expression of several genes essential for limb formation, including those encoding FGFs, Shh and BMPs [17]–[19]. Thus, although double mutant *Sp8 Sp9* mice have not been studied, the *Sp8* single mutant suggests a critical role for these genes in vertebrate limb development.

Here, we use a new deletion of *Sp1* and *btd* that allows us to unambiguously analyze the function of these genes in *Drosophila*. Most strikingly, the complete absence of *Sp1* and *btd*, but not *btd* alone, results in the loss of all leg structures and can lead to a dramatic transformation of leg into wing and notum fates, representing a complete ventral to dorsal fate change. These phenotypes are rescued by resupplying *Sp1*, but not *btd*, suggesting that *Sp1* plays an essential and early role in leg specification. Consistent with these severe phenotypes, early loss of both genes leads to the loss of expression of the telopodite genes *Dll* and *dachshund* (*dac*). However, in contrast to previous findings [14], appendage primordia, as assessed by *esg* expression, still form in the absence of *btd* and *Sp1*. We also find that, like Btd, ectopic expression of Sp1 and vertebrate Sp8 can induce ectopic leg development in dorsal appendages, suggesting that these functions have been conserved. If, however, *btd* and *Sp1* are removed after *Dll* expression is initiated, ventral appendage fates still form, but leg growth is severely compromised. Together, these results suggest that *Sp1* functions upstream of *Dll* and that it plays an early and essential selector-like function in the specification of ventral appendage and body wall fates. Later in development, both *btd* and *Sp1* work in parallel with *Dll* to control the growth and morphology of the legs.

## Results

### Generation of a *btd Sp1* deficiency

**Figure 1:**
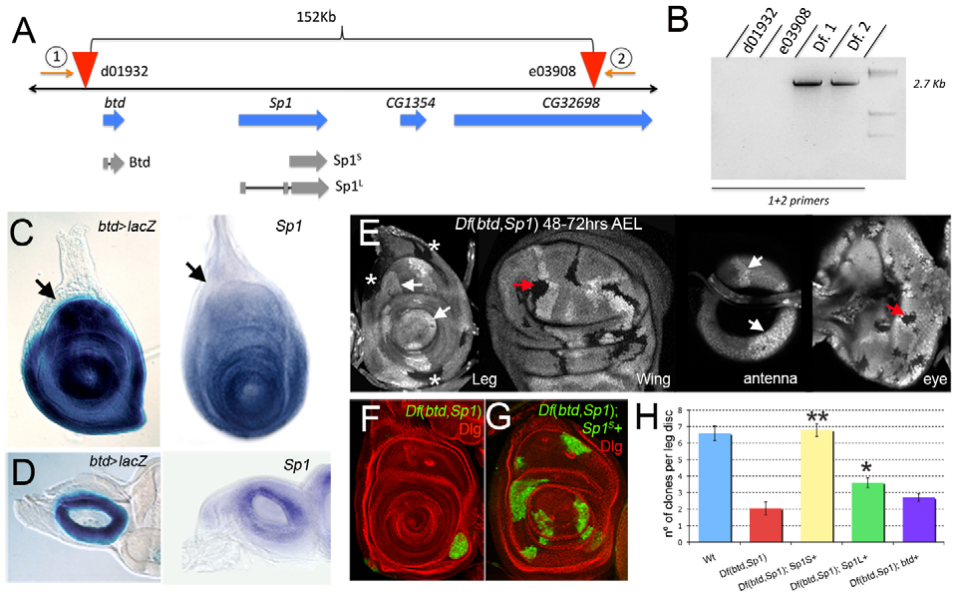
Generating a deficiency for *btd* and *Sp1.* (A) Genomic organization of the *btd* and *Sp1* genomic region. While *btd* only encodes for one isoform, *Sp1* encodes for two, a small one (Sp1-PB (Flybase) or Sp1^S^,) and a larger one (Sp1-PD (Flybase) or Sp1^L^). Two FRT-containing P elements, d01932 and e03908, are situated 5′ and 3′ of *btd* and *Sp1,* respectively. The PCR primers used to molecularly confirm the deficiency are indicated (1 and 2). (B) PCR confirmation of *Df*(*btd,Sp1*). Using the primers shown in (A), no product is observed in the original P element stocks, while a 2.7 kb product is observed in two independently generated *Df*(*btd,Sp1*) stocks. (C,D) *btd* and *Sp1* expression patterns visualized by *btd*-*Gal4*>*UAS*-*lacZ* and *Sp1 in situ* hybridization, respectively, in third instar leg (C) and antennal (D) imaginal discs. Note the absence of *btd* or *Sp1* expression in the presumptive body wall of the leg (arrows). (E) *Df*(*btd,Sp1*) mutant clones (absence of signal) are difficult to recover in the *btd/Sp1* expression domain when they generated before the third instar (<72 hrs AEL). Twin spots (white arrows) and clones in proximal regions (asterisks) can be observed, as can clones in the wing or eye discs (red arrows). Only twin spots are recovered in the medial antenna (white arrows). The images of the antenna and eye discs represent two different confocal planes of the same disc. (F) *Df*(*btd,Sp1*) clones generated 48–72 hrs AEL positively marked by ß -Gal staining (green) in the leg survive poorly and tend to segregate from the surrounding tissue. The disc is co-stained for Discs large (Dlg) which labels all cell membranes (red). (G) MARCM *Df*(*btd,Sp1*); *Sp1^S^*+ mutant clones generated in parallel to those in (F) are recovered more frequently than *Df*(*btd,Sp1*) mutant clones, indicating rescue. (H) Quantification of rescue. *Sp1* rescued the number of clones in the leg disc (only telopodite clones were scored). Note that Sp1^S^ rescued better then Sp1^L^. Clones were induced 48–72 hrs AEL. The rescue experiments with *Sp1^S^* or *Sp1^L^*, but not with *btd+* (p>0.05), show a statistically significant difference from the control experiment (*Df*(*btd,Sp1*); * p<0.05 and ** p<0.001 with Student's t-test).

Although *btd* null alleles are available, there were no mutations that eliminated both *btd* and *Sp1* that could be used for clonal analysis, thus precluding a definitive assessment of the role these genes play in adult development. The distance between *btd* and *Sp1* is approximately 32 kilobases (kb), with no known intervening genes (Figure 1A). To generate a deficiency that removes both *btd* and *Sp1* we used the FRT-directed recombination technique [20]. Using this method, we were able to generate a deficiency that deletes *btd*, *Sp1*, and two adjacent genes with unknown function (CG1354 and CG32698) (Figure 1A; see Materials and Methods). The generation of this deficiency, hereafter referred to as *Df*(*btd,Sp1*), was confirmed by the polymerase chain reaction (PCR) using primers that flank the FRT-containing P elements (Figure 1B).

In third instar imaginal discs, *btd* and *Sp1* have indistinguishable expression patterns as assessed by *in situ* hybridization and by the expression of an enhancer trap inserted close to the transcription start of *btd* (Figure 1C). Both genes were expressed in the entire leg imaginal disc except for the most proximal ring of cells (Figure 1C). Based on its relationship to other markers in the leg, these genes appear to be expressed in the entire leg (coxa through tarsus), but not in the body wall (see Figure S2). Thus, unlike all previously described genes, the *btd* and *Sp1* expression domain marks the tissue that will become leg as opposed to body wall. In the antennal disc, both genes were expressed in a medial ring of cells along the PD axis (Figure 1D). No expression was observed in wing, haltere, or eye discs. Consistent with these expression patterns, mitotic clones of *Df*(*btd,Sp1*) initiated during the second instar or before survived poorly in most of the leg disc (except from the most proximal domain, which gives rise to the body wall), but were readily recovered in wing, haltere, and eye imaginal discs (Figure 1E and 1F). When we examined the cell death marker Caspase 3 (Cas3), we found that clones in the dorsal imaginal discs (eye, wing, and haltere) had no Cas3 staining, while the few clones that survive in the ventral discs (antenna and leg) express Cas3, suggesting that cell death is occurring in these clones (see Figure S3). Consistent with this observation, when *Df*(*btd,Sp1*) clones also expressed the baculovirus cell death inhibitor p35, their growth was partially rescued (see Figure S4).

Because *Df*(*btd,Sp1*) removes two additional genes in addition to *btd* and *Sp1*, we used RNAi knockdown and rescue experiments to address which genes were responsible for the poor survival of *Df*(*btd,Sp1*) clones. Knocking down the expression of the other two genes deleted in *Df*(*btd,Sp1*) using RNAi produced no phenotype in the legs or antennae, suggesting that their absence does not contribute to the poor survival of these clones (data not shown). Using a rescue approach, we tested two different isoforms of *Sp1* (*Sp1^S^* and *Sp1^L^*; Figure 1A) and *btd*. The recovery of *Df*(*btd,Sp1*) clones in the leg disc was rescued to wild type by *Sp1^S^* and, to a lesser degree, by *Sp1^L^* (Figure 1G and 1H). Importantly, the weak rescue provided by *btd* was not statistically significant (Figure 1H). Together with the data described below, these results suggest that the poor survival of *Df*(*btd,Sp1*) clones is largely due to the loss of *Sp1*.

To gain additional insights into the compromised growth of *Df*(*btd,Sp1*) clones, we tested which growth-promoting pathways might be able to rescue this phenotype. Co-expressing *string* (*cdc25*) and *cyclinE*, which promote the cell cycle by promoting the G2 to M transition [21], failed to provide any rescue of *Df*(*btd,Sp1*) clones (see Figure S4). In contrast, expressing the transcriptional co-activator Yorkie (Yki), a downstream component of the Hippo tumor suppressor pathway [22], [23], was able to rescue the growth of *Df*(*btd,Sp1*) clones, both in the leg imaginal disc and the adult leg (see Figure S4). These data suggest that Yki, which is known to activate genes required for proliferation and cell survival [22], [23], functions downstream of Sp1 to activate growth-promoting target genes.

These results show that *Df*(*btd,Sp1*) is a valuable tool to analyze the role of *btd* and *Sp1* during *Drosophila* development. They further suggest that *Sp1* plays a more critical role in leg development than *btd*, a conclusion that we further support below.

### Removing *btd* and *Sp1* functions during larval development results in leg growth defects

**Figure 2:**
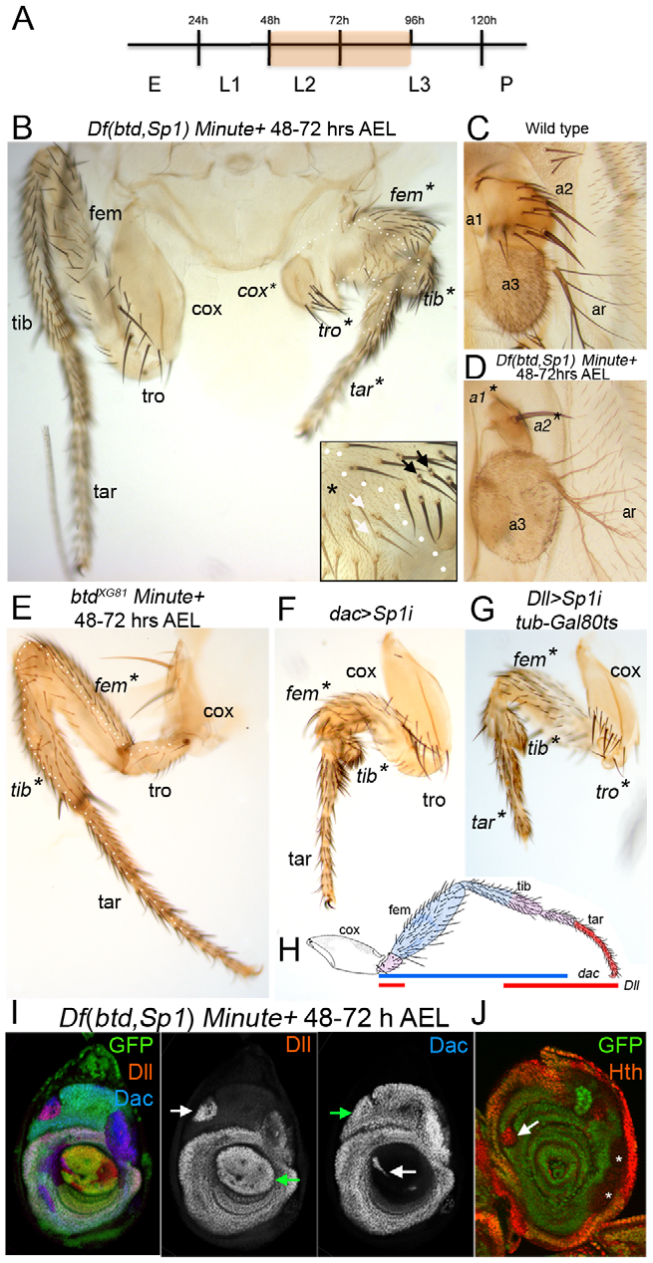
*btd* and *Sp1* control leg growth. (A) Time line showing when removing the function of *btd* and *Sp1* affects leg growth (orange shadow). (B) A large *Df*(*btd,Sp1*) *M+* clone in a T1 leg induced 48–72 hrs AEL and marked by *yellow* (*y*) bristles (the clone boundary is indicated by the white dotted line in the left leg). For comparison, the wild type right T1 leg is included in this image. The mutant tissue still maintains leg identity scored by the presence of bracted bristles (arrows, inset). Asterisks mark the segments affected by the clone. The same leg segment nomenclature has been used for all the figures: coxa (cox), trochanter (tro), femur (fem), tibia (tib) and tarsus (tar). (C) Wild type antenna with 1^st^ antennal segment (a1), 2^nd^ antennal segment (a2), 3^rd^ antennal segment (a3) and arista (ar). (D) *Df*(*btd,Sp1*) *M+* clone induced 48–72 hrs AEL results a strong reduction in size of the a1 and a2 antennal segments, while a3 and the ar are normal. The clone is marked by *y*. (E) A large *btd^XG81^ M+* clone in a T2 leg induced 48–72 hrs AEL and marked by *y* (clone is outlined by white dots) results in a small growth defect in the femur (fe) and tibia (tib), which are also partially fused (arrow). The tarsus (tar), trochanter (tro) and coxa (cox) are unaffected. (F, G, H) The downregulation of *Sp1* beginning at the second instar using RNAi affects the growth of the entire leg. Two different Gal4 drivers were used to examine different regions of the leg. (F) The medial part of the leg is strongly reduced in size in *dac*-*Gal4*; *UAS*-*Sp1i* flies. (G) The distal part of the leg is strongly reduced in size in *Dll*-*Gal4*; *UAS*-*Sp1i* flies. In this experiment we blocked Gal4 activity prior to the second instar using *tub-Gal80^ts^*. (H) Shows a schematic representation of the expression patterns of the two Gal4 drivers used to downregulate *Sp1* function (*dac* in blue and *Dll* in red). (I, J) *Df*(*btd,Sp1*) *M+* clones induced 48–72 hrs AEL and examined in 3^rd^ instar leg discs. (I) A subset of *Df*(*btd,Sp1*) *M+* clones (marked by the absence of GFP) show de-repression of *Dll* in the Dac domain and de-repression of *dac* in the Dll domain (white arrows). Note that these clones do not affect the expression of *Dll* and *dac* in their normal expression domains (green arrows). (J) A subset of *Df*(*btd,Sp1*) *M+* clones (marked by the absence of GFP) show derepression of *hth* (arrow). Clones that do not derepress *hth* are indicated with asterisks.

To assess the role these genes play at different times during development, we first analyzed the behavior of *Df*(*btd,Sp1*) clones in the adult that were induced in the second instar stage (48 to 72 hrs after egg laying (AEL)), long after the imaginal discs have been allocated and *Dll* expression has been initiated. To give these clones a growth and survival advantage, we used the *Minute* (*M*) technique, which allows the generation of tissue comprised of entirely, or almost entirely, homozygous mutant cells [24]. No phenotypes were observed in the dorsal appendages (wing or haltere) or dorsal body (see below). In contrast, legs containing *Df*(*btd,Sp1*) *M+* clones were dramatically reduced in size (Figure 2B). Growth defects were observed throughout the entire leg, from the coxa to the tip of the tarsus. However, leg identity, assessed by the presence of bracted bristles, was still maintained in *Df*(*btd,Sp1*) tissue (Figure 2B, inset). When recovered in the antenna, the sizes of the a1 and a2 segments were also severely reduced, while the a3 and arista segments were unaffected (Figure 2C and 2D), consistent with the expression of *btd* and *Sp1* in a medial ring in the antennal imaginal disc (Figure 1D). These findings suggest that, by 48 hrs of development, neither *btd* nor *Sp1* are required to maintain leg identities, but that one or both of these genes is required for proper leg growth and morphology.

To determine which of these two genes is required for leg growth at this stage we examined the effects of eliminating or knocking down *btd* and *Sp1* individually. Large *btd^XG81^ M+* clones made between 48 to 72 hrs AEL only generated weak phenotypes in the femur and tibia, which were partially fused (Figure 2E). These results support the idea that *btd* plays only a minor role in leg development. Because a *Sp1* null allele is not available, we improved upon earlier RNAi knockdown experiments [14] to assess the role of *Sp1* (see Materials and Methods). In contrast to the weak phenotypes observed in *btd^XG81^* clones, reducing *Sp1* activity by RNAi resulted in growth defects that were similar to those observed in large *Df*(*btd,Sp1*) clones (Figure 2F–2H). Analogous results were observed in the antenna: *btd^XG81^* clones had no effect, while knockdown of *Sp1* phenocopied the loss of the a1 and a2 segments seen in *Df*(*btd,Sp1*) mutant clones (see Figure S5). Taken together, these results suggest that *Sp1* is playing a more important role than *btd* in leg and antennal growth after 48 hrs AEL.

We next examined the behavior of *Df(btd,Sp1) M+* clones in the leg imaginal discs. For these experiments, we analyzed the expression of the three primary genes expressed along the proximo-distal (PD) axis, *Dll*, *dachshund* (*dac*), and *homothorax* (*hth*). In wild type third instar leg discs, these genes are expressed in overlapping domains along the PD axis to create five unique combinations [25]. From distal to proximal, these combinations are: 1) *Dll* only, 2) *Dll* + *dac*, 3) *dac* only, 4) *Dll* + *dac* + *hth*, and 5) *hth* only [26], [27]. In 86% (n = 23) of the *Df*(*btd,Sp1*) *M+* clones recovered in the *dac* only domain *Dll* was derepressed, without any effect on *dac* expression (Figure 2I). In a smaller number of clones (34%; n = 32), we observed the de-repression of *hth* in the *dac*-only domain (Figure 2J). Similarly, 44% (n = 36) of the *Df*(*btd,Sp1*) *M+* clones recovered in the *Dll* only domain de-repressed *dac*, without affecting *Dll* expression (Figure 2I). No effect on *hth* expression was observed in clones present in the most proximal domain of the leg disc (data not shown). Importantly, the expression patterns of *Dll, dac,* and *hth* were unaffected in *btd^XG81^ M+* clones (data not shown). These data demonstrate that *Sp1* plays an important role in generating the unique domains of gene expression that comprise the leg's PD axis.

### Removing *btd* and *Sp1* function during embryogenesis results in severe leg truncations

Previous work using a much larger deficiency (*Df(1)C52*) that removes *btd*, *Sp1*, and >50 other genes suggested that *btd* and *Sp1* are required for the embryonic expression of *Dll* [14]. Using *Df*(*btd,Sp1*), we find that *Dll* expression is barely detectable in the leg primordia of stage 15 embryos (see Figure S6). In addition, in contrast to what was previously suggested based on the larger deficiency, formation of the leg primordia, as monitored by *escargot* (*esg*) expression, does not require *btd* and *Sp1*, because *esg* expression was still observed, although reduced, in *Df*(*btd,Sp1*) homozygous embryos (see Figure S6). As will be described below, the weak residual Dll protein that is observed in older *Df*(*btd,Sp1*) embryos is likely due to the activity of the early *Dll-304* enhancer, which does not require *btd* or *Sp1* inputs.

The near absence of *Dll* expression in older embryos contrasts with the relatively subtle effects on *Dll* expression when *btd* and *Sp1* activities are removed 48 hrs AEL or later (Figure 2I and data not shown). One possible scenario to reconcile this difference is that *btd* and *Sp1* have two temporally distinct functions during leg development: early, during embryogenesis, they would be required to maintain or perhaps establish ventral appendage fates, in part by activating *Dll*. Later in development, during larval stages, they would only be required for the proper growth of the leg.

**Figure 3:**
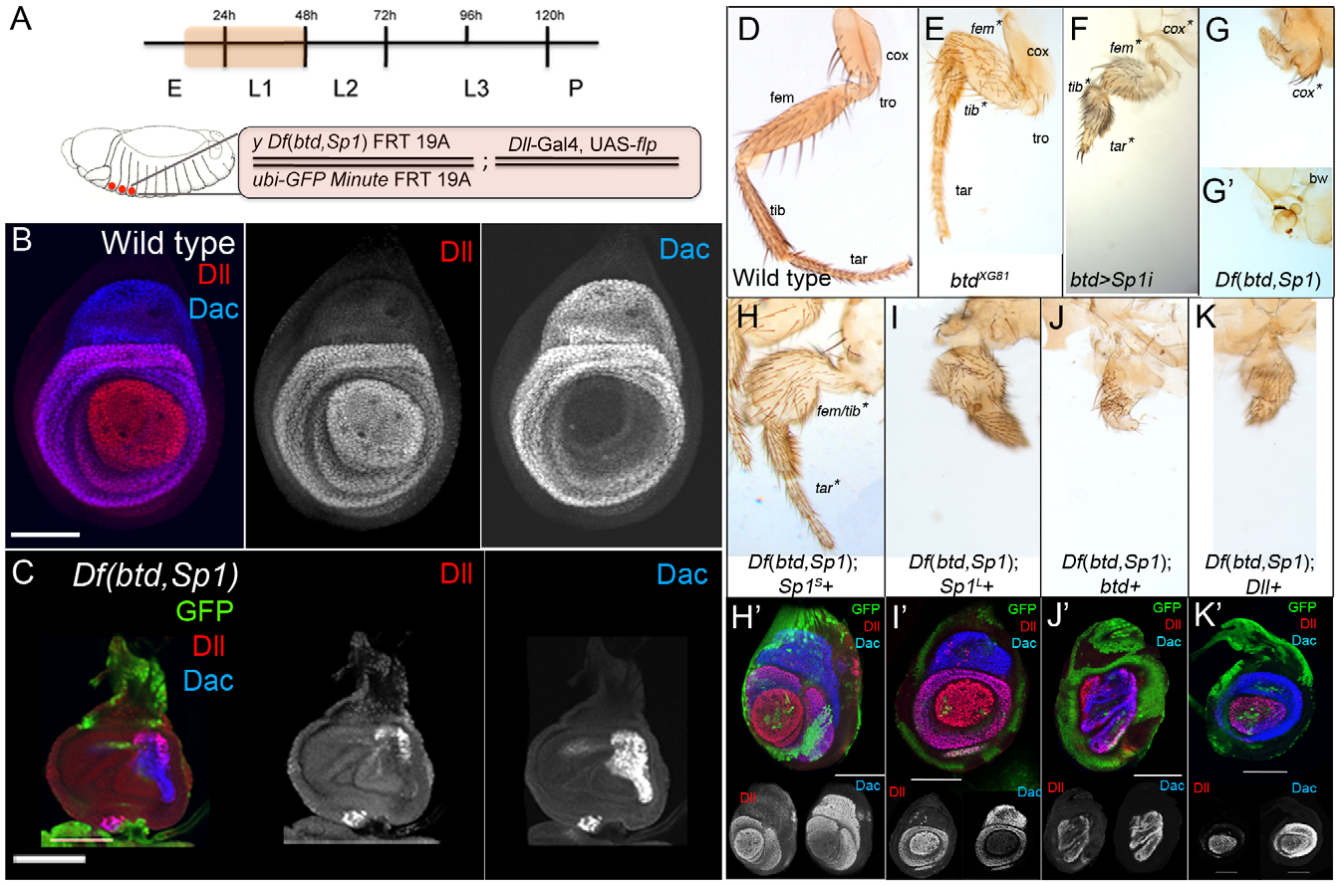
*Sp1* is required for leg development. (A) Top: time line showing when the functions of *btd* and *Sp1* (orange shadow) were removed. Bottom: In these experiments we initiated clone induction during embryogenesis using *Dll*-*Gal4*; *UAS*-*flp* to generate *Df*(*btd,Sp1*) *M+* or *btd M+* clones. This method results in excellent survival of animals that have all six legs completely, or nearly completely, mutant. For the rescue experiments, an additional UAS transgene (e.g. *UAS-btd*) was included. (B) Wild type third instar leg imaginal disc showing the expression patterns of *Dll* and *dac*. (C) Third instar *Df*(*btd,Sp1*) mutant leg generated using the genotype schematized in (A); mutant tissue is marked by the absence of GFP. These discs are much smaller than wild type and show a nearly complete loss of *Dll* and *dac* expression. White bar is 75 µm. (D) Wild type T1 adult leg with the segments indicated. (E) T1 adult leg entirely mutant for *btd* (marked by *y*) generated as shown in (A). Only the size of the femur and the tibia are affected and are partially fused together. (F) *btd*-*Gal4*; *UAS*-*Sp1i* reduces the size of the entire leg, from the coxa to the distal tip. Shown here is a T1 leg. (G,G') T1 adult legs entirely (G') or nearly entirely (G) mutant for *btd* and *Sp1*, generated as described in (A). Mutant tissue is marked by *y*. In (G), only a small patched of mutant tissue is visible and is associated with some non-mutant (*y+*) coxa tissue. In (G'), no leg tissue is observed. (H–K) Rescue of *Df*(*btd,Sp1*) T1 mutant legs (marked by *y*) generated as described in (A) where (H) *Sp1^S^*, (I) *Sp1^L^*, (J) *btd* and (K) *Dll* were expressed under the control of *Dll*-*Gal4*. (H'-K') show examples of leg imaginal discs of the same genotypes. White bars represents 75 µm. (H) *Sp1^S^* is able to rescue the *Df*(*btd,Sp1*) mutant phenoype and restore the *Dll* and *dac* expression domains. (I) *Sp1^L^* is able to partially rescue the adult *Df*(*btd,Sp1*) mutant phenoype and completely restore the *Dll* and *dac* expression domains. (J) *btd* is unable to rescue the adult *Df*(*btd,Sp1*) mutant phenoype and partially rescues the *Dll* and *dac* expression domains. (K) *Dll* is unable to rescue the adult *Df*(*btd,Sp1*) mutant phenoype but partially rescues *Dll* and *dac* expression domains.

To test this idea, we analyzed the behavior of *Df*(*btd,Sp1*) *M+* clones generated during embryogenesis. Using *Dll-Gal4; UAS-flp* to induce mitotic recombination (see Materials and Methods), 100% of the adults had severely aberrant legs. In 90% of the adults, the legs were completely absent or consisted of only a small patch of residual leg tissue (Figure 3G). When leg tissue was observed, it was invariably associated with non-mutant tissue, suggesting that these clones were generated slightly later than those samples in which no leg tissue remained. Generating *btd^XG81^* clones at this early time produced relatively minor fusions of the femur and tibia, but left the tarsal segments largely unaffected (Figure 3E). These phenotypes are similar to those observed in later-induced *btd* clones (compare with Figure 2E). In contrast, reducing *Sp1* activity by RNAi resulted in severe defects throughout the entire leg (Figure 3F). These results suggest that *Sp1* is playing a more important role than *btd*, a conclusion that is supported by rescue experiments. When *btd+* was resupplied in the *Df*(*btd,Sp1*) *M+* legs, the resulting appendages were still highly abnormal, indicating poor rescue (Figure 3J). In contrast, when *Sp1^S^* or, to a lesser degree, *Sp1^L^*, were resupplied in *Df*(*btd,Sp1*) *M+* legs, significant rescue of leg development was observed (Figure 3H and 3I).

Similar conclusions come from the analysis of leg imaginal discs containing *Df*(*btd,Sp1*) *M+* clones generated during embryogenesis. Because these clones were generated early, entirely mutant leg discs could be obtained. In most cases, the discs were greatly reduced in size, with no or little *Dll* expression, and only a small patch of residual *dac* expression (Figure 3C). Strikingly, the normal expression domains of *Dll* and *dac* could be fully rescued by resupplying *Sp1^S^* or *Sp1^L^* (Figure 3H' and 3I'). In contrast, resupplying *btd* to *Df*(*btd,Sp1*) *M+* discs provided a very weak rescue of these PD expression domains (Figure 3J'). Together, these experiments suggest that *Sp1* is required during embryogenesis to generate leg fates, while *btd* plays a much more restricted role in leg development. Similarly, as noted above, *Sp1* plays a much more important role in antennal development than *btd* (see Figure S5).

### Ventral to dorsal transformations in the absence of *btd* and *Sp1*

**Figure 4:**
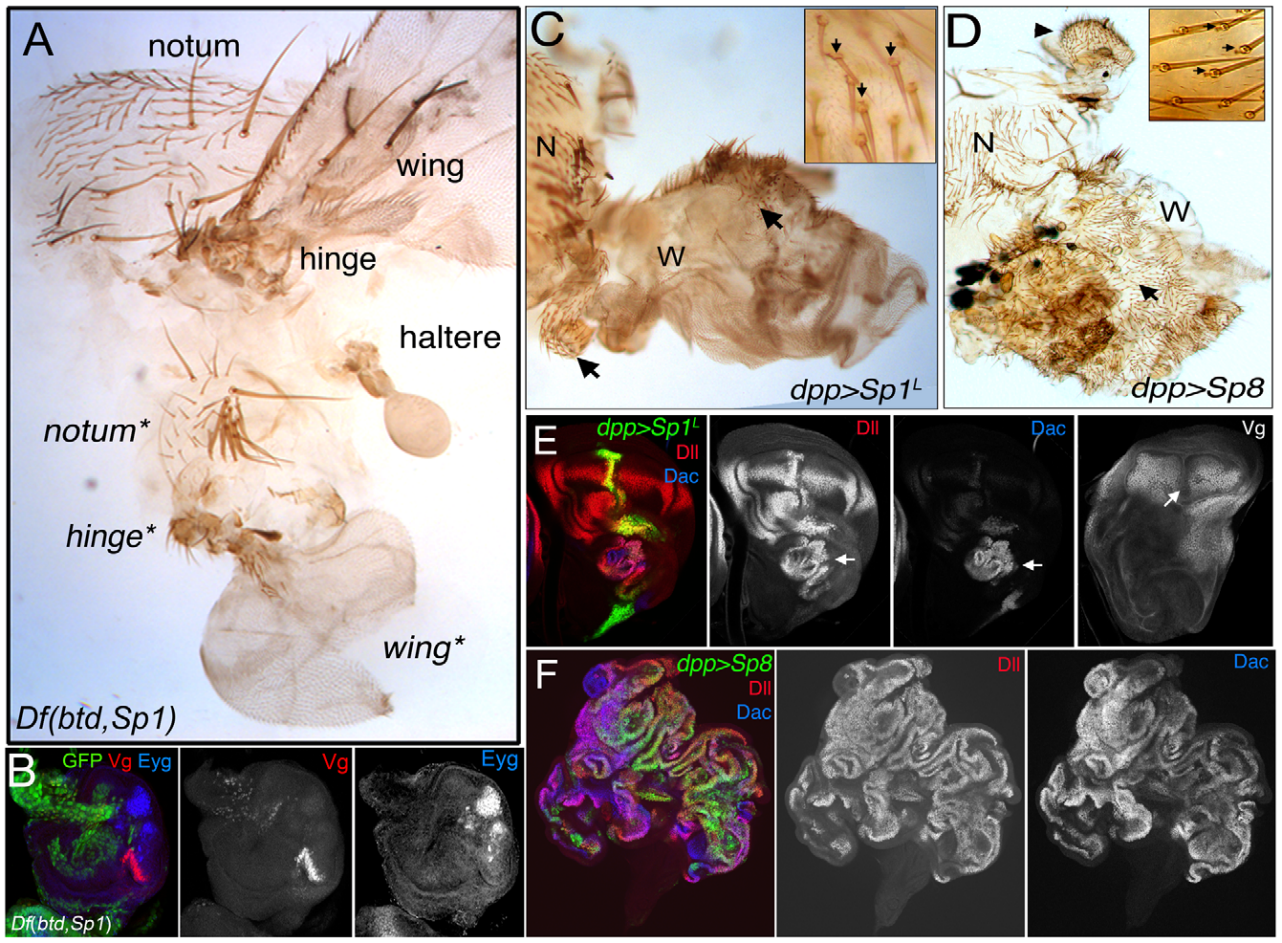
Dorsal to ventral transformations resulting from the loss of *btd* and *Sp1*. (A) A T2 adult segment comprised mostly of *Df*(*btd,Sp1*) *y* tissue. These animals are generated via the genotype shown in Figure 3A. Dorsal is up. The normal notum, wing, and hinge are at the top; the bottom half of the tissue shows a transformation of ventral fates towards dorsal fates, including an ectopic wing, hinge, and notum (indicated by asterisks). Note that the normal notum, wing, and hinge are mutant (marked by *y*) but appear wild type. (B) Third instar leg imaginal disc of the same genotype as in (A) stained for GFP (absence marks the mutant tissue), Vg (red) and Eygone (Eyg; blue), which are markers for wing and notum fates, respectively. (C,D) Ectopic expression of *Sp1^L^* (C) or mouse *Sp8* (D), under the control of *dpp*-*Gal4* result in the transformation of wing towards leg in the adult. Arrows indicate leg tissue. Remaining notum (N) and wing (W) tissue are indicated. Insets show a high magnification of the leg tissue, with bracts (small arrows). Note the appearance of leg structures also in the pronotum in (E) (arrowhead). (E,F) Ectopic expression of *Sp1^L^* (E) or mouse *Sp8* (F), under the control of *dpp*-*Gal4* results in the induction of leg fates in the wing imaginal disc. These discs were stained for *dpp-Gal4* expression (green), Dll (red), Dac (blue), or Vg (E, right-most panel). *dpp>Sp8* also results in dramatic overgrowths that are not observed in the *dpp>Sp1^L^* wing discs.

In addition to observing severely truncated or no legs, in about 10% of the adults with *Dll>flp* induced *Df*(*btd,Sp1*) *M+* clones one or two of the legs were transformed towards a dorsal thoracic fate, including elements of the wing or haltere, and notum (Figure 4A and Table S1). In some examples we observed the triple row of bristles characteristic of the dorso-ventral border of the wing blade (data not shown). These ventral to dorsal homeotic transformations were confirmed by the presence of wing and notum molecular markers in mutant *Df*(*btd,Sp1*) leg discs, including *vestigial* (*vg*) and *eyegone* (*eyg*) (Figure 4B) [28], [29]. In addition to observing wing tissue, we also observed haltere tissue in place of the third thoracic legs in some of these animals (Figure S7 and Table S1). Curiously, these ventral to dorsal transformations did not always respect the normal thoracic identities because wing tissue, which normally develops in the second thoracic (T2) segment, was frequently observed in the T1, T2, and, to a lesser extent, the T3 segments (see Table S1). Nevertheless, these dramatic transformations indicate that *btd* and *Sp1* are required for establishing adult ventral fates and that they inhibit the establishment of dorsal fates. Ventral to dorsal transformations were never observed when only *btd* function was removed at this early time, arguing that *Sp1* is sufficient for executing these selector-like gene functions.

Ectopic expression experiments also support the idea that *Sp1* behaves as a ventral appendage selector gene. Using either flip-out Gal4 (not shown) or *dpp-Gal4* (Figure 4), *Sp1^L^* was able to activate both *Dll* and *dac* and inhibit *vg* expression in the wing imaginal disc and produce wing to leg transformations in the adult appendage (Figure 4C and 4E). Ectopic expression of *Sp1^L^* was also able to induce another proximal leg gene, *teashirt* (*tsh*), in the wing, in a pattern that was reminiscent of that seen in wild type leg discs (see Figure S8). Moreover, this property has been evolutionarily conserved in this gene family because mouse *Sp8* can also activate *Dll* and *dac* and induce dramatic dorsal to ventral homeotic transformations (Figure 4D and 4F).

### *Sp1* is required for Dll-LT, but not Dll-304, activity

To examine the connection between *btd, Sp1,* and *Dll* at higher resolution, we analyzed the dependencies of two *Dll* enhancers, *Dll-304* and *Dll-LT*, on *btd* and *Sp1* activities. *Dll-LT* is directly activated by Wg and Dpp inputs [30]. Consequently, a *LT-lacZ* reporter gene is expressed in the center of the leg imaginal disc, in cells that receive strong input from both of these signaling pathways [30], [31]. One question that stems from these previous studies is why *Dll-LT* activity is specific to the ventral appendages and is not activated in other tissues where Wg and Dpp activities intersect such as the wing disc. The data described above suggest that *btd* and/or *Sp1* may be the answer.

**Figure 5:**
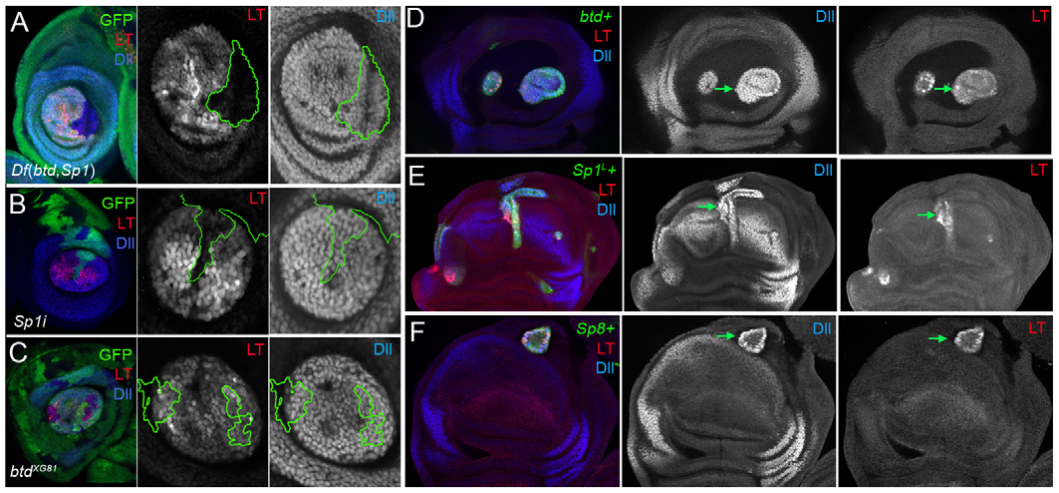
*Sp1*, not *btd*, is required for *Dll-LT* activity. (A) *Df*(*btd,Sp1*) *M+* clone (outlined in green) generated 72–96 hrs AEL shows the absence of *LT-lacZ* expression, but no affect on *Dll*. (B) Clones expressing *Sp1i* strongly reduced *LT-lacZ* expression. (C) *btd^XG81^* mutant clones generated 72–96 hrs AEL do not affect *LT-lacZ* expression. (D–F) Ectopic expression of *btd* (D), *Sp1^L^* (E), or mouse *Sp8* (F) in the wing disc activates *Dll* and *LT-lacZ* (green arrows). These flip-out clones were generated 48–72 hrs AEL.

To test this idea, we generated *Df*(*btd,Sp1*) *M+* clones and analyzed the effects on the *LT-lacZ* reporter gene in leg imaginal discs. Strikingly, *LT-lacZ* expression was absent in these clones (Figure 5A). This appears to be a consequence of the loss of *Sp1* and not *btd* because *btd^XG81^* clones had no effect on *LT-lacZ* expression (Figure 5C). Further, the down regulation of *Sp1* by RNAi was sufficient to strongly reduce, but not eliminate, *LT-lacZ* expression (Figure 5B). Note that, consistent with our earlier studies [30], no effect on *Dll* expression was observed in these clones because the maintenance of *Dll* expression in larval stages is independent of *Sp1* and *btd* (Figure 5A–5C). Ectopic expression of *Sp1* also induced the expression of *LT-lacZ* and *Dll* in the wing disc (Figure 5E). *LT-lacZ* and *Dll* can also be activated by mouse *Sp8* and by *btd* (Figure 5D and 5F). Thus, although *btd* is not required for *LT* activity, it has the capacity to induce its activity when ectopically expressed. This gain-of-function property of *btd* is consistent with previous observations that *btd* is sufficient to induce leg development when ectopically expressed in the wing [14].

**Figure 6:**
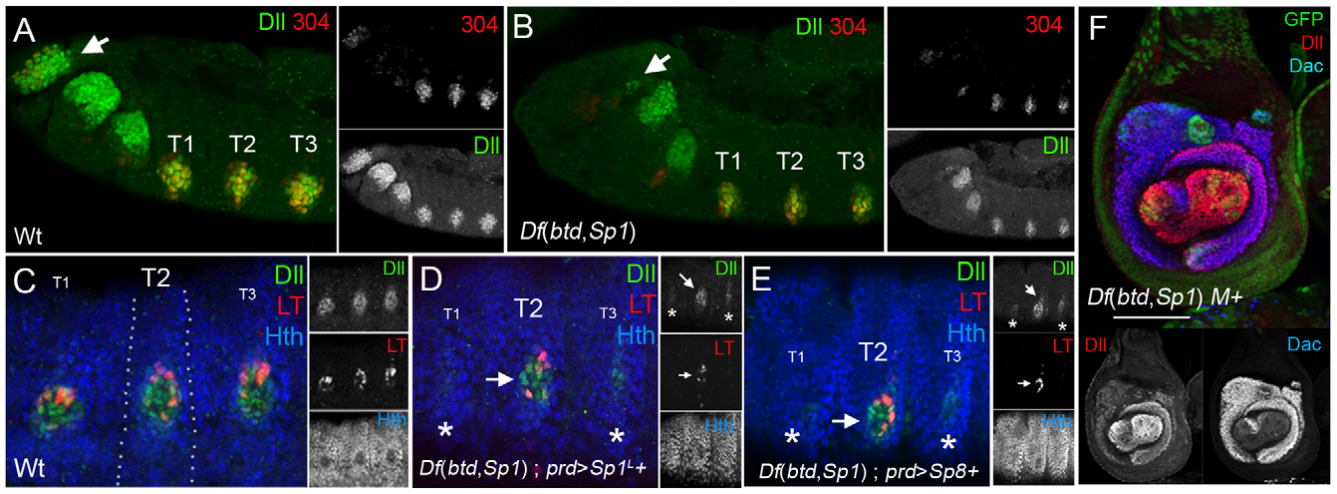
Different dependencies on *Sp1* for early and late *Dll* enhancer activities. (A,B) Stage 11 wild type (A) and *Df*(*btd,Sp1*) (B) embryos stained for Dll (green) and *Dll304-lacZ* (red). *Dll304-lacZ* remains active in the absence of both factors. Expression of *Dll* in the antennal primordia, however, is nearly absent in *Df*(*btd,Sp1*) embryos (arrows). T1, T2, and T3 mark the three thoracic segments. (C) Wild type stage 14 embryo stained for Dll (green), Hth (blue), and *Dll-LT-lacZ* (red). The white dots mark the *prd-Gal4* expression domain. (D, E) *Df*(*btd,Sp1*); *prd-Gal4*; *UAS*-*Sp1^L^* (D) or *UAS-Sp8* (E) stage 14 embryos. In T1 and T3, where *prd-Gal4* is not expressed, *Dll* expression is greatly reduced (asterisks) and LT activity is completely absent. The remaining *Dll* expression is likely derived from the *Dll304* early enhancer. In T2, where *prd>Sp1^L^* (D) or *prd>Sp8* (E) both *Dll* and *LT-lacZ* expression are rescued. *btd* and *Sp1^S^* can also rescue embryonic *Dll* and *LT-lac*Z expression (not shown). (F) *Df*(*btd*,*Sp1*) *M+* mutant leg disc generated using the scheme shown in Figure 3A, rescued with *UAS-Sp8*. Significant rescue of the *Dll* (red) and *dac* (blue) expression domains is observed. Mutant tissue is marked by the absence of GFP (green).

*LT-lacZ* is first activated in stage 14 embryos [2]. Consistent with the above findings, *Df*(*btd,Sp1*) embryos failed to express *LT-lacZ* (Figure 6D). In contrast, *LT-lacZ* is expressed in *btd* embryos, although at reduced levels [2]. The lack of *LT-lacZ* expression in *Df*(*btd,Sp1*) embryos could be rescued by resupplying only *Sp1* (Figure 6D). Remarkably, mouse *Sp8* was also able to rescue *Dll* expression and *LT* activity in *Df*(*btd,Sp1*) embryos (Figure 6E) and *Dll* and *dac* expression in *Df*(*btd,Sp1*) mutant leg imaginal discs (Figure 6F). In contrast to *Dll-LT*, the earlier-acting *Dll* enhancer, *Dll-304*, did not require *btd* or *Sp1* because a *304-lacZ* reporter gene was expressed in *Df*(*btd,Sp1*) embryos (Figure 6B). The independence of *Dll-304*, but dependence of *Dll-LT*, on *Sp1* activity accounts for the observation that *Df*(*btd,Sp1*) stage 14 embryos show very weak, residual Dll protein in the leg primordia (Figure 6D and 6E).

### *btd* and *Sp1* function upstream of Dll

**Figure 7:**
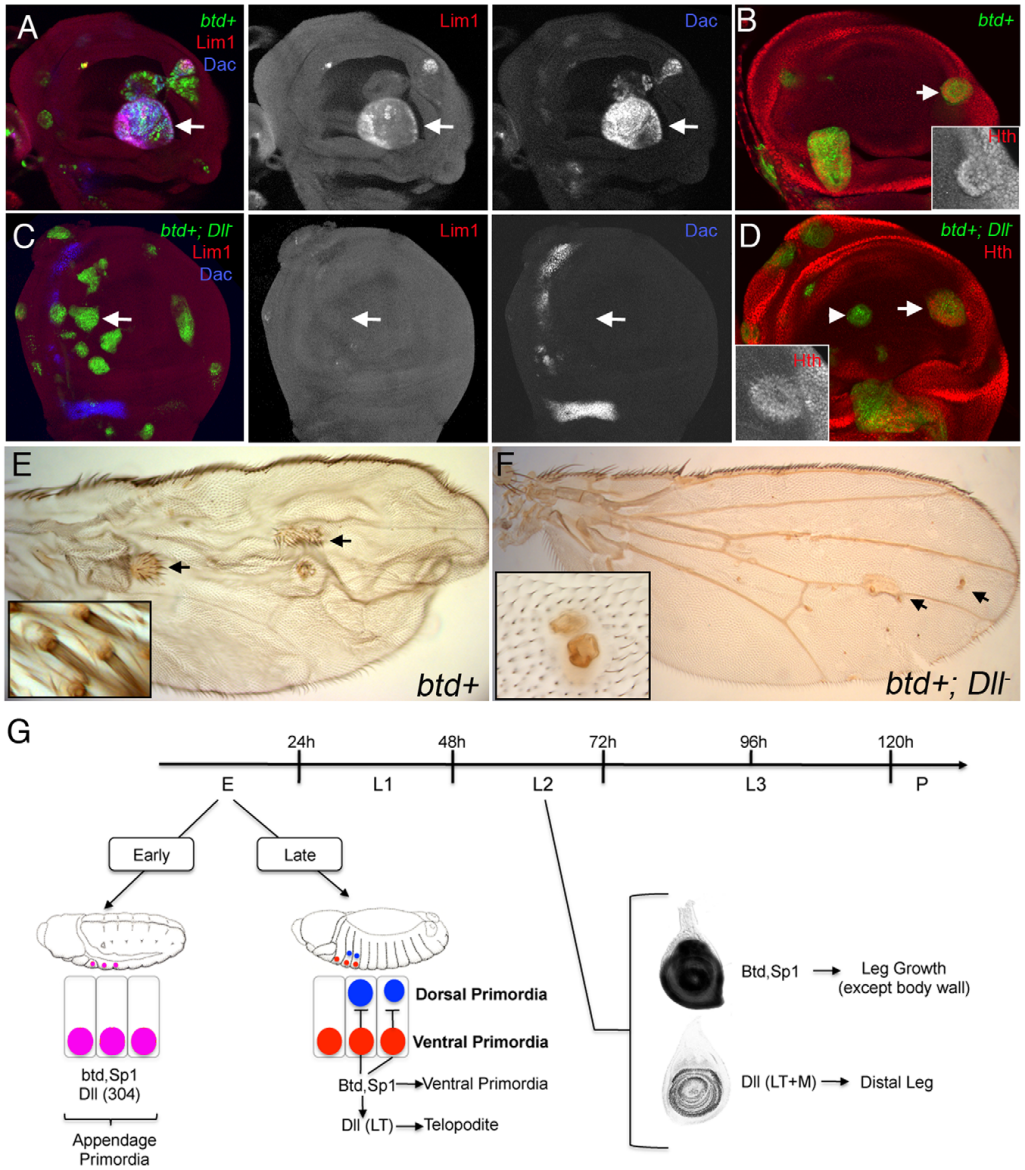
*btd* and *Sp1* require *Dll* to induce distal and medial leg development. (A,B) *btd+* MARCM clones in the wing disc activated the expression of *Lim1*, *dac* (A) and *hth* (B) (arrows). Clones were generated 48–72 hrs AEL and are marked by GFP+ (green). (C,D) *Dll-*; *btd+* MARCM clones in the wing disc were unable to induce *Lim1* or *dac* (C; arrows), but can still able activate *hth* close to its own domain (arrow) but not in the center of the wing pouch (D; arrowhead). Clones were generated 48–72 hrs AEL and are marked by GFP (green). (E) *btd+* MARCM clones in the adult wing blade induced the formation of leg-like tissue, including distal leg identities (arrows and inset). (F) *Dll*-; *btd*+ MARCM clones failed to induce distal leg-like tissue, although they generate tissue that might correspond to proximal leg tissue (arrows and inset). (G) Schematic representation of the differential requirements for *btd* and *Sp1* during leg development. At embryonic 11 stage, *Dll* (via the *304* enhancer), *btd*, and *Sp1* are all activated independently in the appendage primordia. A few hours later, *Dll* expression is restricted to the telopodite precursors cells of the leg (via the *LT* enhancer) and depends on *Sp1* activity. At this stage, *Sp1* is required to promote the formation of the ventral appendage primordia (legs) and inhibit the formation of the dorsal primordia (wing and haltere). *Dll* is required for the entire telopodite domain. During larval second instar stage (L2), *Dll* expression no longer requires *btd* and *Sp1*. *Dll* is required only for distal leg development while *btd* and *Sp1* are required for the growth of the entire leg but have no function in the body wall.

As described above, *btd* and *Sp1* both have the ability to induce ectopic leg development when expressed in the dorsal imaginal discs, and both have the ability to induce *Dll* expression. Given our observation that the initiation of LT activity is also dependent on *btd* and *Sp1*, we reasoned that the ability of these factors to induce leg development, especially distal leg fates, might depend on *Dll*. To test this, we used the MARCM method [32] to generate clones that ectopically express *btd* or *Sp1^L^* and at the same time were mutant for *Dll* (*tub>btd; Dll^–^* or *tub>Sp1^L^; Dll^–^*). In control *tub>btd* clones (wild type for *Dll*), ectopic leg tissue was observed in the wing, and markers for leg development (*Lim1, dac,* and *hth*) were activated in the wing imaginal disc (Figure 7A and 7B). In contrast, when these clones were also mutant for *Dll*, the activation of *Lim1* and *dac*, which are markers for the distal leg, was not observed (Figure 7C). However, *tub>btd; Dll^–^* clones close to the wing hinge were still able to activate *hth*, a marker for proximal fates (Figure 7D). Similar observations were obtained in *tub>Sp1^L^; Dll^–^* clones (see Figure S9). From these data, we conclude that *btd* requires *Dll* to generate the *Lim1+, dac+* telopodite, but that *btd* can induce proximal, *hth+*, leg fates in the absence of *Dll*. This conclusion is further supported by the behavior of *tub>btd+; Dll^–^* clones that arise in the adult wing. Although these clones cannot generate distal leg fates, they are able to produce what appears to be proximal leg tissue (Figure 7F). In contrast, *tub>btd* clones have the ability to induce both proximal and distal leg structures in the adult wing (Figure 7E). Finally, the epistatic relationship between *btd*, *Sp1*, and *Dll* was further supported by analyzing the consequences of resupplying *Dll+* in *Df*(*btd,Sp1*) *M+* legs and leg discs. The resulting legs were still severely truncated, indicating poor rescue, while in the imaginal discs *Dll* and *dac* expression was only partially rescued (Figure 3K). These phenotypes likely reflect the later requirement of *Sp1* and, to a lesser extent, *btd*, in leg growth and cell survival (see above).

In summary, *btd* and *Sp1* have the capacity to induce telopodite fates, which depend on *Dll*, as well as more proximal coxapodite fates, which do not require *Dll*.

## Discussion

Prior to this study, our understanding of the roles that *btd* and *Sp1* play in ventral appendage development in *Drosophila* was largely derived from ectopic expression experiments showing that *btd* could induce ectopic leg development when expressed in dorsal imaginal discs [14]. In addition, based on a large deficiency that removes >50 genes, it was suggested that these genes may function upstream of *Dll* in ventral appendage specification. What was lacking in this previous study was the ability to specifically analyze the functions of these genes, both in embryogenesis and during adult development, using loss-of-function null alleles. Here, using a newly derived deficiency, together with rescue experiments, we show for the first time that these Zn-finger transcription factors play non-redundant roles in ventral appendage development. Moreover, for all of the readouts examined here – leg allocation, leg growth, proliferation, and PD axis formation – *btd* plays a much more minor or no role compared to *Sp1*. Early, *Sp1*, but not *btd*, is required to define the group of cells that will give rise to the legs and perhaps additional ventral body structures as well. Thus, *Sp1* is a selector-like gene for the entire ventral appendage. Later in development, both genes are required for the proper growth of the leg, although to very different degrees. We also show that vertebrate *Sp8* retains both the selector and growth-promoting functions, suggesting that there has been a remarkable amount of functional conservation between the vertebrate and fly genes. Below we discuss both functions, and summarize how these findings contribute to our overall understanding of ventral appendage development in *Drosophila*.

### Growth-promoting functions of *btd* and *Sp1* during larval stages

During larval development, we find that *Sp1* is required for the proper growth of the entire leg, from the coxa through the tarsus. In contrast, *btd* plays a much more limited role in the tibia and femur. At this stage, neither gene is required for leg identity, nor are they required for the development of ventral body structures that arise from the most proximal cells in the leg imaginal disc. These ‘late’ phenotypes are consistent with the expression patterns of these genes in the third instar leg imaginal discs, where they appear to mark the entire presumptive leg, but not more proximal cells. This is interesting, because prior to these observations there were no markers that distinguished between the *hth*-expressing cells that give rise to the coxa from the *hth*-expressing cells that give rise to the ventral body wall. *Dll*, for example, is expressed in the cells that give rise to the distal tibia and tarsus, and lineage tracing with the *Dll-LT* element marks the entire telopodite (trochanter, femur, tibia, and tarsus) [2]. The addition of the *btd* and *Sp1* expression patterns and mutant phenotypes to previously characterized PD genes therefore adds an important demarcation that distinguishes leg from body fates.

Our analysis also reveals dramatic differences in the post-embryonic functions of *btd* and *Sp1.* Specifically, most of the growth phenotypes observed when both genes are removed can be phenocopied by knocking down only *Sp1*. In contrast, *btd^XG81^* clones (or *btd^XA^* clones, see Materials and Methods) have no phenotypes in the antenna, and, in the leg, result in only partial fusions between the femur and tibia. Thus, *Sp1*, not *btd*, plays an important and non-redundant function in ventral appendage development at this stage.

### Selector-like functions of *Sp1*

Selector and selector-like genes have the property that they specify an entire organ or body part [33]. The classic example is *engrailed* (*en*) which ‘selects’ posterior compartment identities in *Drosophila* [34]. Another example is *eyeless* (*ey*), which is both necessary and sufficient for eye development in *Drosophila* [35]. In the leg, previous work highlighted the role of *Dll* in ventral appendage specification. In the absence of *Dll*, the distal portion of the leg fails to develop, while dorsal appendages remain wild type [1]. Moreover, ectopic expression of *Dll* can induce distal legs to develop in dorsal positions [10]. Taken together, these observations suggested that *Dll* is a selector-like gene for the distal leg.

Despite the requirement for *Dll* in leg development, it has been known for sometime that the ventral appendage primordia form in the absence of *Dll* [1], [9]. Moreover, homeotic transformations are not observed in the absence of *Dll*. Thus, *Dll* cannot be considered a selector-like gene for the entire ventral appendage. These observations raise the question of what factor or factors initially specify the cells that will give rise to the ventral appendage. We propose that *Sp1* fulfills this selector-like role.

The suggestion that *Sp1* is a selector-like gene for the entire ventral appendage stems in part from the observation that when the function of this gene is removed early in development, ∼10% of the animals have dramatic transformations of ventral structures to dorsal structures. In many of these cases, we observe both wing and notum tissue developing in ventral positions. Molecularly, *Dll* and *dac* expression is lost in transformed leg discs, and ectopic expression of *vg* and *eyg*, two markers for the dorsal appendages, are observed instead. The expression of *Dll-304*, which is traditionally been considered a marker for the ventral appendage, in *Df*(*btd,Sp1*) embryos may seem at odds with the idea that *Sp1* is required for the initial specification of leg fates. However, fate-mapping studies show that *Dll-304*-expressing cells give rise to both the ventral (leg) and dorsal (wing and haltere) appendages [2]. Thus, *Dll-304* cannot be considered a ventral marker, and its activity in *Df*(*btd,Sp1*) embryos only confirms the establishment of appendage primordia without ventral or dorsal identity.

In sum, the striking transformations of fate seen in *Df*(*btd,Sp1*) animals suggest that *Sp1* promotes ventral fates, both the entire leg and ventral body wall, and that in the absence of this gene, dorsal fates are de-repressed. This change in developmental fate is analogous to other classical homeotic transformations, for example, when the leg is transformed to antenna in the absence of *Antennapedia* (*Antp*) [36]. Note that *btd* null clones made at the same early time in development only result in mild growth defects, but legs are still generated. Thus, *btd* is not required for this function. However, because an *Sp1* null allele (*btd+*) is not currently available, we cannot at this time be completely certain that *btd* plays no role in this process.

Because wing development is normally limited to T2, it was unexpected to observe leg to wing transformations in the T1 and, to a lesser extent, T3 segments. One potential explanation for this violation of antero-posterior identity is due to the timing of clone induction. Although the Hox genes are responsible for determining the segmental identities of the dorsal appendages [37], [38], it may be that they are deployed at different times in the ventral and dorsal primordia in the different thoracic segments. If this is the case, then the resulting transformations may be very sensitive to the time they were generated and to their segmental origins. It is also worth noting that the wing primordia and T2 identity can be generated in the absence of Hox input [39], [40]. Thus, wing fates, as opposed to haltere or humeral (dorsal T1) fates, represent a Hox-free default state, which may predominate in these aberrant developmental situations.

### Gradual refinement of ventral fates

Together with previous studies, these findings allow us to present a more complete view of ventral appendage specification, which we breakdown into three main phases (Figure 7G). In the first phase, *Sp1*, *btd*, and *Dll* (via it’s early *Dll-304* enhancer) are initially activated in parallel in a ventral domain in each thoracic hemisegment of stage 11 embryos. The activation of all three genes is dependent on Wg signaling [1], [14]. This early, *Dll-304*-driven expression of *Dll* does not require either *btd* or *Sp1*. This initial group of cells is fated to give rise to both the entire ventral and dorsal thoracic imaginal discs, in other words, the entire adult thorax. In the second phase, which begins at stage 14, *Dll-304* is no longer active and *Dll* is controlled by late-acting enhancers such as *Dll-LT*, which is activated by Wg and Dpp signaling [2], [30]. Interestingly, as shown here, these late-acting *Dll* enhancers also require *Sp1*, but not *btd* [2], thus placing *Sp1* genetically upstream of *Dll*. At this stage, the *Dll+* cells will only give rise to the leg telopodite. *Sp1* is also required for telopodite formation but is carrying out at least two additional functions. One is that, unlike *Dll*, *Sp1* is required to specify more proximal leg segments (the coxapodite). Second, the ventral to dorsal homeotic transformations described above suggest that *Sp1* is also required to repress dorsal fates. Finally, in the third phase, *Dll* begins to autoactivate it’s expression and no longer depends on Wg and Dpp inputs [30], [41]. At this stage, *Dll* also no longer requires *Sp1* to be expressed. Instead of working through *Dll*, *btd* and *Sp1* continue to play a critical role in leg development but now work in parallel to *Dll* to promote the growth of the entire leg. Thus, the specification of the ventral primordia depends on a feed-forward logic in which *Sp1* activates late embryonic *Dll* expression followed by a phase in which both *btd* and *Sp1* contribute to appendage growth in parallel to *Dll* (Figure 7G).

### Appendage development: a case of “deep homology”

Besides having a PD axis, arthropod and vertebrate appendage morphologies have little in common. Moreover, the developmental logic of limb formation in *Drosophila* is very different from that of vertebrate limb development. In flies, Hedgehog signaling induces two antagonistic secondary signals, Dpp and Wg, which in turn establish the PD axis by activating genes such as *Dll* and *dac* [41], [42]. In vertebrate limb development, Sonic hedgehog induces the activity of fibroblast growth factor-like molecules such as FGF8 in the ectoderm, which drives the proliferation of the underlying mesenchyme and the nested expression of Hox genes to create a PD axis [43], [44]. Despite these differences, it is striking that multiple vertebrate orthologs of both *Sp1* and *Dll* are expressed during vertebrate limb development. In addition, orthologs of both *hth* and *exd* (*Meis* and *pbx*, respectively) are expressed in the proximal domain of the developing mouse limb [45], [46]. Although the existence of multiple *Dll* and *Sp1* orthologs (*Dlx1/Dlx2/Dlx5/Dlx6* and *Sp8/Sp9*, respectively) makes it much more challenging to assess their functions in detail, the available data demonstrate that, as in flies, both sets of genes are critical for vertebrate limb development [17]–[19], [47], [48]. Our results, illustrating that vertebrate *Sp8* can rescue many of the *Sp1* and *btd* loss of function phenotypes in *Drosophila*, support the idea that appendage development in these two phyla represents a case of ‘deep homology’ [49], [50]. Interestingly, that orthologs of both *Sp1* and *Dll* gene families are used in both phyla argue that, for appendage development, the functions of these transcription factors have been much more conserved than those of the signaling pathways used in limb development. The same conclusion holds for eye development where the transcription factors, more than the deployment of specific signaling pathways, have been conserved over vast evolutionary distances [49], [51]. These observations imply that, once established, transcription factor networks may be very stable, while the organization of signaling pathway networks may be much more plastic and easily modified to accommodate radically distinct morphologies.

## Materials and Methods

### Generation of the *Df*(*btd,Sp1*)

To generate *Df*(*btd,Sp1*) we used the FRT-directed recombination technique using two FRT-containing P elements (PBac{XP}d01932 and PBac{RB}CG32698^e03908^). Recombinants lose the *miniwhite* gene, providing a positive identification for the recombination event. Two independent deletions were generated and confirmed by PCR using primers flanking the genomic region or within the P elements. Besides *btd* and *Sp1* this deletion also removes *CG1354* (molecular function: GTP binding) and partially deletes *CG32698* (molecular function: carbonate dehydratase activity) (DrosDel FDD-0029282, http://www.drosdel.org.uk).

### Generation of *UAS*-*Sp1^L^* and mouse *UAS*-*Sp8*

*Sp1^L^* and *Sp1^S^* are called *Sp1-RD* and *Sp1-RB*, respectively, by FlyBase (http://flybase.org). For the *UAS*-*Sp1^L^* construct we isolated RNA from leg imaginal discs to generate cDNA (SuperScript III First Strand Synthesis System for RT-PCR, Invitrogen). This served as a template to amplify the Sp1^L^ isoform by PCR, which was sequenced and cloned into the pUAST attB vector. For mouse *UAS*-*Sp8* we cloned the mouse *Sp8* cDNA (gift from A. Mansouri) into a 3XHA-tagged pUAST attB vector.

### Fly stocks

Two *btd* mutations were studied, the strong *btd^XG81^* mutation and the amorph *btd^XA^* [52]. We found that *btd^XG81^* phenotypes are stronger than *btd^XA^*, in agreement with Cohen and Jurgens [52]. To knock down *Sp1* function, we combined two *UAS-RNAi* hairpin transgenes, one described by Estella et al. [14] and one from the Vienna Drosophila Resource Center (VDRC; line #4097). The Vienna RNAi stock is reported to have no off-target affects. As confirmation of this, we only observed phenotypes in tissues where *Sp1* is expressed. Both transgenes, which target both *Sp1* isoforms, were used in conjunction with *UAS*-*dicer* to enhance the RNAi, and thus generated much stronger phenotypes than were previously reported [14]. *Dll ^SA1^* [8], *UAS*-*Dll* [10], *UAS*-*btd* [13], *Dll*-*Gal4* line 212; [10], *btd*-*Gal4* [14], *dac*-*Gal4* [53], and *prd*-*Gal4* [54] have been described. The *dpp*-*Gal4*; *UAS*-*GFP*, *tub-Gal80^ts^* and *UAS*-*flp* were from the Bloomington Stock Center. The *UAS*-*Sp1* was from [13] was renamed *UAS*-*Sp1^S^* because it encodes the short *Sp1* isoform. The two Dll elements, *Dll-304-lacZ* [8] and *LT-LacZ* [14] were described. *ubi-GFP* FRT19A; *hs-flp* and *ubi-GFP M(1)^osp^* FRT19A were gifts from G. Struhl. *UAS-yki* was from D.J. Pan [55] and *UAS-p35, UAS-string*, and *UAS-cycE* were from L. Johnston.

### Clonal analysis

To generate these genotypes we used a duplication on the Y chromosome that covers the *btd* and *Sp1* genes (*Dp*(*1;Y*)*lz^+^*) [13].

-btd *clones*

*yw btd^XG81^* or *btd^XA^* FRT19A/*ubi*-*GFP M(1)^osp^* FRT19A; *Dll-Gal4*, *UAS-flp* or *hs-flp*.

-Df(btd,Sp1) *loss of function clones*

*yw Df(btd,Sp1)* FRT19A/*ubi*-*GFP M(1)^osp^* FRT19A; *Dll-Gal4, UAS-flp* or *hs-flp*.

*yw Df(btd,Sp1)* FRT19A/yw ubi-GFP FRT19A; *hs-flp*

Because *Minute/+* flies are developmentally delayed by approximately 1 day we adjusted the time of the heat-shock to induce clones at the correct developmental stage. Larvae were heat shocked for 1 hour at 37°C.

btd, Sp1^S^, Sp1^L^, *and* Sp8 *gain of function clones*.

*yw hs-flp; act>y+>Gal4 UAS-GFP.* The larvae were heat shocked for 10 minutes at 37°C.

Dll-; UAS-btd or UAS-Sp1^L^ *MARCM clones*.

*yw hs-flp*, *UAS-GFP*; FRT42D y+ *tubG80/Dll^Sa1^* FRT 42D; *tub-Gal4*

Df(btd,Sp1); UAS-btd, UAS-Sp1^S^ *or* UAS-Sp1^L^ *MARCM clones.*

*tubGal80* FRT19A/*Df(btd,Sp1)* FRT19A; *tub-Gal4*, *UAS-lacZ*

Df(btd,Sp1); UAS-btd, UAS-Sp1^S^ *or* UAS-Sp1^L^, *or* UAS-Sp8 *rescue experiments.*

*yw Df(btd,Sp1)* FRT19A/*ubi-GFP M(1)^osp^* FRT19A; *Dll-Gal4*, *UAS-flp*

### Immunofluorescence methods

Imaginal discs and embryos were prepared and stained using standard procedures. RNA *in situ* hybridizations were carried out with digoxigenin-labeled RNA probes against *btd* and *Sp1* [14]. For the *Sp1* probe, the first and second exons of *Sp1^L^* were cloned in pBSK and transcribed to generate the anti-sense probe. These exons partially overlap a non-coding exon of *Sp1^S^*, so is likely to hybridize to both *Sp1* transcripts. The primary antibodies used were: rabbit and mouse anti-ßGal (Capell and Promega), rabbit anti-GFP (Invitrogen), mouse anti-Dachsund and mouse anti-Dlg (Developmental Studies Hybridoma Bank (DSHB)), rabbit anti-caspase-3 (Upstate biotechnologies), guinea pig anti-Distalles, rabbit anti-Homothorax, rat anti-Lim1 (gift from Gerard Campbell), guinea pig anti-Vestigial (gift from M. Zecca) and rabbit anti-Vestigial (gift from Sean Carroll) and guinea pig anti-Eyegone (gift from N. Azpiazu).

## Supporting Information

Figure S1 *btd* and *Sp1* are expressed in the leg primordia. Embryos are oriented anterior to the left and dorsal up. *Sp1* (A) and *btd* (B) RNA *in situ* hybridization in stage 13 embryos reveals the expression of these genes in the leg primordia (arrows). The inset at the right show a higher magnification image of the thoracic segments.
(0.89 MB TIF)

Figure S2 *btd-Gal4* is expressed in the coxa but not in the body wall. (A) Third instar imaginal disc stained for Dll (blue), Hth (red) and GFP (*btd-Gal4; UAS-GFP*). Three different views of the same imaginal disc are shown with the most proximal domains marked. Note that at this stage *btd* is expressed at low level is the trochanter (tro), strongly in the coxa (cox) but is not expressed in the body wall (bw). (B) Schematic representation of the imaginal disc shown in (A). Note that *btd* is expressed in the entire leg at different levels but is not expressed in the body wall. (C) Everting pupal leg disc stained as in (A). The double-headed arrow indicates the PD axis of the leg. Note that *btd* expression subdivides the *hth* expression domain into presumptive coxa (*btd+ hth+*) and body wall (*btd- hth+*).
(2.53 MB TIF)

Figure S3 *btd* and *Sp1* mutant clones activate cell death. *Df*(*btd,Sp1*) positively marked (ß-Gal, green) mutant clones generated 48–72hrs are readily recovered in the wing (A) or eye (C) discs, while in the leg discs (B) or second segment of the antenna disc (D) are rarely recovered and tend to segregate from the surrounding tissue. When recovered, these clones activate the apoptotic program as indicated by the expression of the cell death marker Cas 3 (red). These discs were stained with Dlg (blue) to identify cell membranes. The small panels in (A) and (B) show optical cross-sections of the *Df*(*btd,Sp1*) clones in the wing and leg discs, respectively. The eye-antenna imaginal disc shown in (C) and (D) is the same disc imaged in different confocal planes.
(3.80 MB TIF)

Figure S4 *yki* rescue of *Df*(*btd,Sp1*) mutant clones. (A) MARCM *Df*(*btd,Sp1*) clones generated 48-72 hrs AEL positively marked by b-Gal staining (red) in the leg imaginal disc survive poorly. The disc is co-stained for Hth which labels the proximal domain of the leg (green). (B) MARCM *Df*(*btd,Sp1*); *yki*+ mutant clones generated in parallel to those in (A) are recovered more frequently than *Df*(*btd,Sp1*) mutant clones, indicating rescue. (C) Adult leg resulting from the same experiment as in (A). Note the nearly absence of *Df*(*btd,Sp1*) mutant tissue marked by *yellow* (*y*). The arrow points to one clone that has sorted out form the main epithelium. (D) Adult leg resulting from the same experiment as in (B). Note that providing Yki in *Df*(*btd,Sp1*) mutant clones can rescue the appearance of mutant clones (arrows, marked by *y*). The inset shows a mutant clone that has sorted out form the main tissue but maintains a leg identity. (E) Quantification of rescue. *yki*, and to a lesser extent *p35*, rescued the number of clones in the leg disc (only telopodite clones were scored). Note that *stg* + *cyclin-E* do not rescue. Clones were induced 48-72 hrs AEL. Each column shows the mean and standard error of the mean. All three independent experiments ((*Df* (*btd,Sp1*) plus *p35*, *yki* or *stg* and *cyclin-E*) are different from (*Df* (*btd,Sp1*) mutant clones (* p<0.05,** p<0.001 with Student's t-test).
(5.54 MB TIF)

Figure S5 *Sp1,* but not *btd,* is required for antennal growth. All antennae are labeled with: 1^st^ segment (a1), 2^nd^ segment (a2), 3^rd^ (a3) and arista (ar). (A) Wild type antenna. (B) Completely *btd^XG81^* mutant antenna marked by *y* of the geneotype: *yw btd^XG81^* FRT19A/*ubi-GFP M* FRT19A; *Dll-Gal4*, *UAS-flp*. No phenotype is observed in the mutant antenna. (C) *btd*-*Gal4*; *UAS*-*Sp1i* reduces the size of the a1 and a2 antennal segments. Compare to (A). (D) Antenna of the genotype *yw Df*(*btd,Sp1*) FRT19A/*ubi-GFP M* FRT19A; *Dll-Gal4*, *UAS-flp* where the a1 and a2 segments are greatly reduced.
(1.34 MB TIF)

Figure S6 *btd* and *Sp1* mutant embryos fail to maintain *Dll* expression. Thoracic regions of stage 14 embryos stained for ß-Gal (*esg-LacZ*, green) and Dll (red). Anterior is left and dorsal is up. (A) Wt embryo showing the thoracic appendage primordia (legs, wing and haltere primordia). (B) *Df*(*btd,Sp1*) mutant embryo that fails to maintain *Dll* expression, compare it to (A).
(0.71 MB TIF)

Figure S7 Ventral to dorsal transformation in the absence of *btd* and *Sp1.* Hemi-third thoracic segment of a fly of the genotype *yw Df*(*btd,Sp1*) FRT19A/*ubi-GFP M* FRT19A; *Dll-Gal4*, *UAS-flp* where the third leg is transformed to an haltere (asterisks). Dorsal is to the left and ventral is to the right.
(0.62 MB TIF)

Figure S8 Ectopic expression of *Sp1* induces leg development in the wing disc. (A) *dpp-Gal4; UAS-Sp1^L^* induces the ectopic expression of the leg PD genes *Dll* (green), *dac* (blue), and *tsh* (red) in the wing imaginal disc. Two planes of focus are shown. Note that the tissue where *Dll*, *dac* and *tsh* are ectopically induced (white square) is organized as a wild type leg imaginal disc. (B) A wild type leg imaginal disc shown for comparison.
(0.90 MB TIF)

Figure S9 *Sp1* requires *Dll* to induce leg development. (A) *Sp1^L^* ectopic expression clones in the wing disc induce the expression of the leg genes *dac* and *tsh* (arrows). Clones are generated 48-72 hrs AEL. Note that Sp1 is better able to induce *dac* expression in the notum that in the wing pouch. (B) *Dll-*; *Sp1^L^+* MARCM clones fail to induce *dac* expression (arrows). However, these clones retain the ability to activate *tsh.* Clones are generated 48-72 hrs AEL.
(1.76 MB TIF)

Table S1 Summary of ventral to dorsal transformations. We scored the number of animals of the genotype *yw Df*(*btd,Sp1*) FRT19A/*ubi-GFP M* FRT19A; *Dll-Gal4*, *UAS-flp* that had a leg transformation to a dorsal appendage (wing or haltere) in any of the three thoracic segments. The ambiguous category includes those animals that had dorsal transformations but could not be unambiguously scored as wing-like or haltere-like. Total number of animal counted  =  41.
(0.08 MB TIF)
