# Supplementary figures and images for: Non-Redundant Selector and Growth-Promoting Functions of Two Sister Genes, buttonhead and Sp1, in Drosophila Leg Development
Source: PLoS Genet. 2010 Jun 24;6(6):e1001001. doi: 10.1371/journal.pgen.1001001 (PMC2891808; doi:10.1371/journal.pgen.1001001)

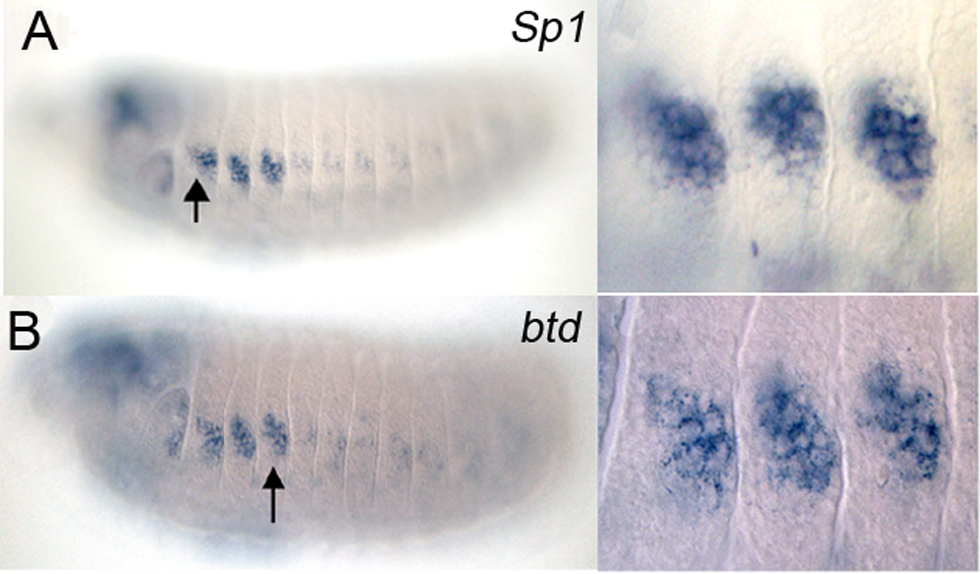

Supplement: Figure S1 — btd and Sp1 are expressed in the leg primordia. Embryos are oriented anterior to the left and dorsal up. Sp1 (A) and btd (B) RNA in situ hybridization in stage 13 embryos reveals the expression of these genes in the leg primordia (arrows). The inset at the right show a higher magnification image of the thoracic segments. (0.89 MB TIF) [file pgen.1001001.s001.tif]

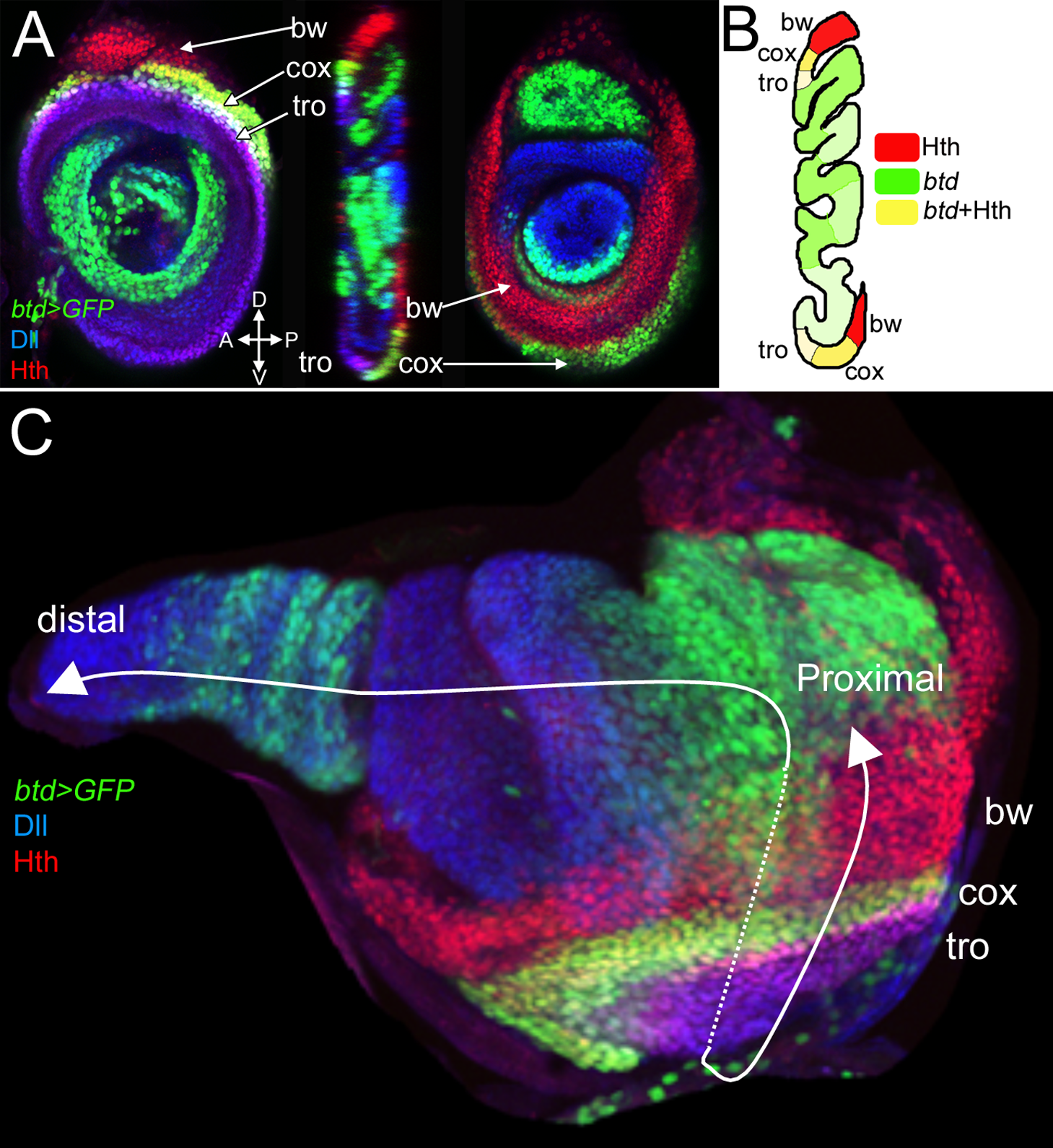

Supplement: Figure S2 — btd-Gal4 is expressed in the coxa but not in the body wall. (A) Third instar imaginal disc stained for Dll (blue), Hth (red) and GFP (btd-Gal4; UAS-GFP). Three different views of the same imaginal disc are shown with the most proximal domains marked. Note that at this stage btd is expressed at low level is the trochanter (tro), strongly in the coxa (cox) but is not expressed in the body wall (bw). (B) Schematic representation of the imaginal disc shown in (A). Note that btd is expressed in the entire leg at different levels but is not expressed in the body wall. (C) Everting pupal leg disc stained as in (A). The double-headed arrow indicates the PD axis of the leg. Note that btd expression subdivides the hth expression domain into presumptive coxa (btd+ hth+) and body wall (btd- hth+). (2.53 MB TIF) [file pgen.1001001.s002.tif]

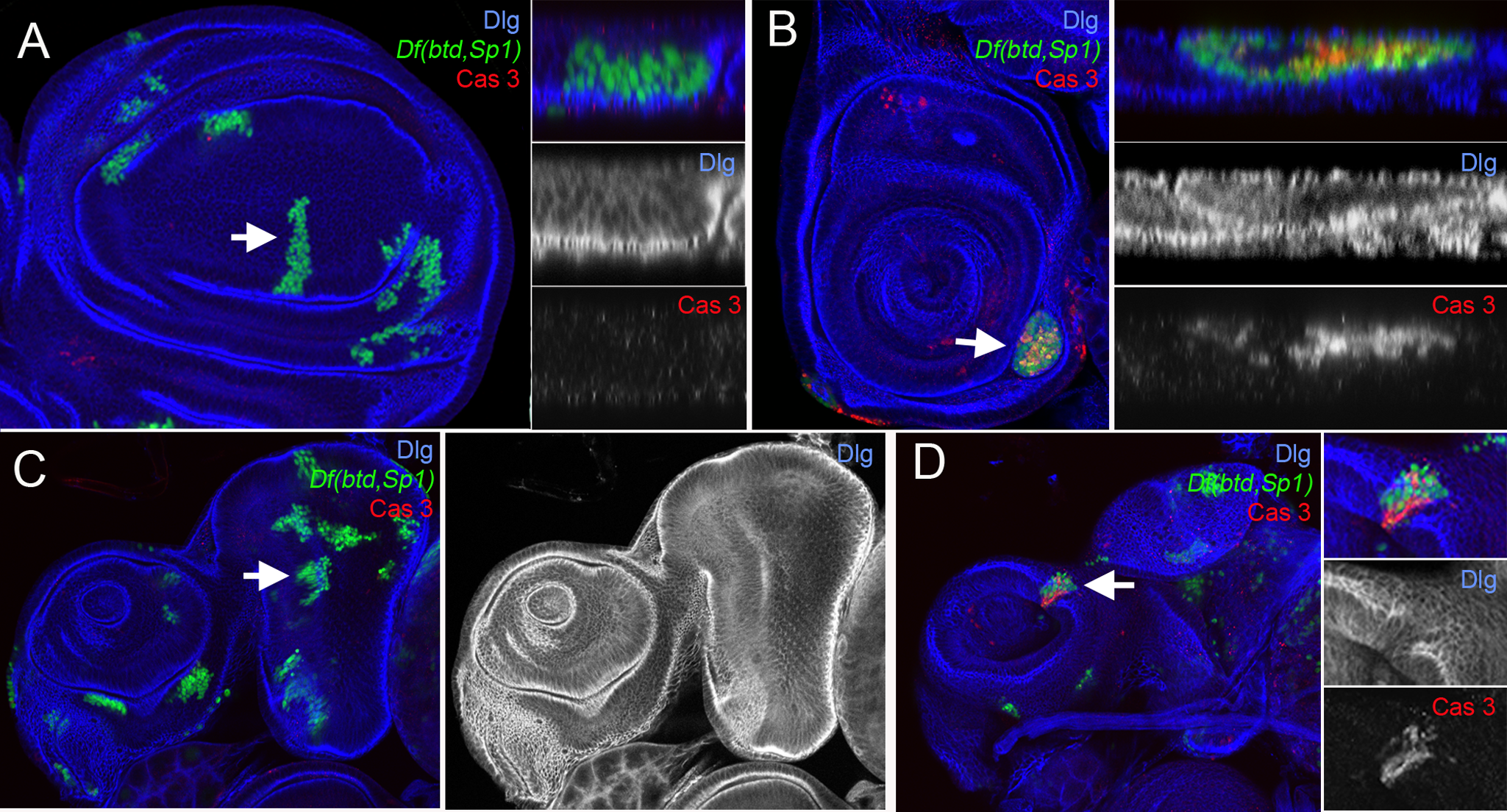

Supplement: Figure S3 — btd and Sp1 mutant clones activate cell death. Df(btd,Sp1) positively marked (ß-Gal, green) mutant clones generated 48–72hrs are readily recovered in the wing (A) or eye (C) discs, while in the leg discs (B) or second segment of the antenna disc (D) are rarely recovered and tend to segregate from the surrounding tissue. When recovered, these clones activate the apoptotic program as indicated by the expression of the cell death marker Cas 3 (red). These discs were stained with Dlg (blue) to identify cell membranes. The small panels in (A) and (B) show optical cross-sections of the Df(btd,Sp1) clones in the wing and leg discs, respectively. The eye-antenna imaginal disc shown in (C) and (D) is the same disc imaged in different confocal planes. (3.80 MB TIF) [file pgen.1001001.s003.tif]

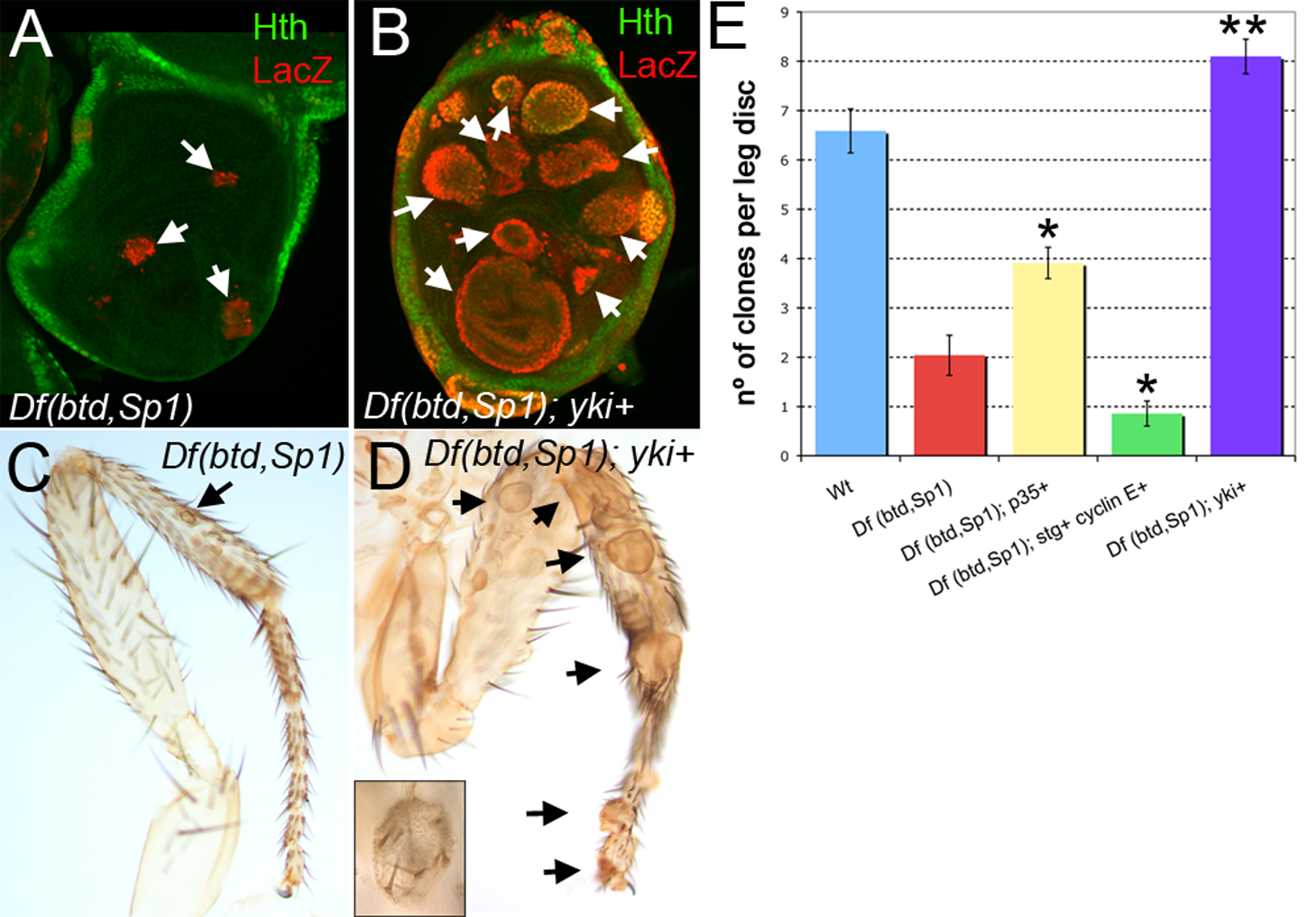

Supplement: Figure S4 — yki rescue of Df(btd,Sp1) mutant clones. (A) MARCM Df(btd,Sp1) clones generated 48-72 hrs AEL positively marked by b-Gal staining (red) in the leg imaginal disc survive poorly. The disc is co-stained for Hth which labels the proximal domain of the leg (green). (B) MARCM Df(btd,Sp1); yki+ mutant clones generated in parallel to those in (A) are recovered more frequently than Df(btd,Sp1) mutant clones, indicating rescue. (C) Adult leg resulting from the same experiment as in (A). Note the nearly absence of Df(btd,Sp1) mutant tissue marked by yellow (y). The arrow points to one clone that has sorted out form the main epithelium. (D) Adult leg resulting from the same experiment as in (B). Note that providing Yki in Df(btd,Sp1) mutant clones can rescue the appearance of mutant clones (arrows, marked by y). The inset shows a mutant clone that has sorted out form the main tissue but maintains a leg identity. (E) Quantification of rescue. yki, and to a lesser extent p35, rescued the number of clones in the leg disc (only telopodite clones were scored). Note that stg + cyclin-E do not rescue. Clones were induced 48-72 hrs AEL. Each column shows the mean and standard error of the mean. All three independent experiments ((Df (btd,Sp1) plus p35, yki or stg and cyclin-E) are different from (Df (btd,Sp1) mutant clones (* p<0.05,** p<0.001 with Student's t-test). (5.54 MB TIF) [file pgen.1001001.s004.tif]

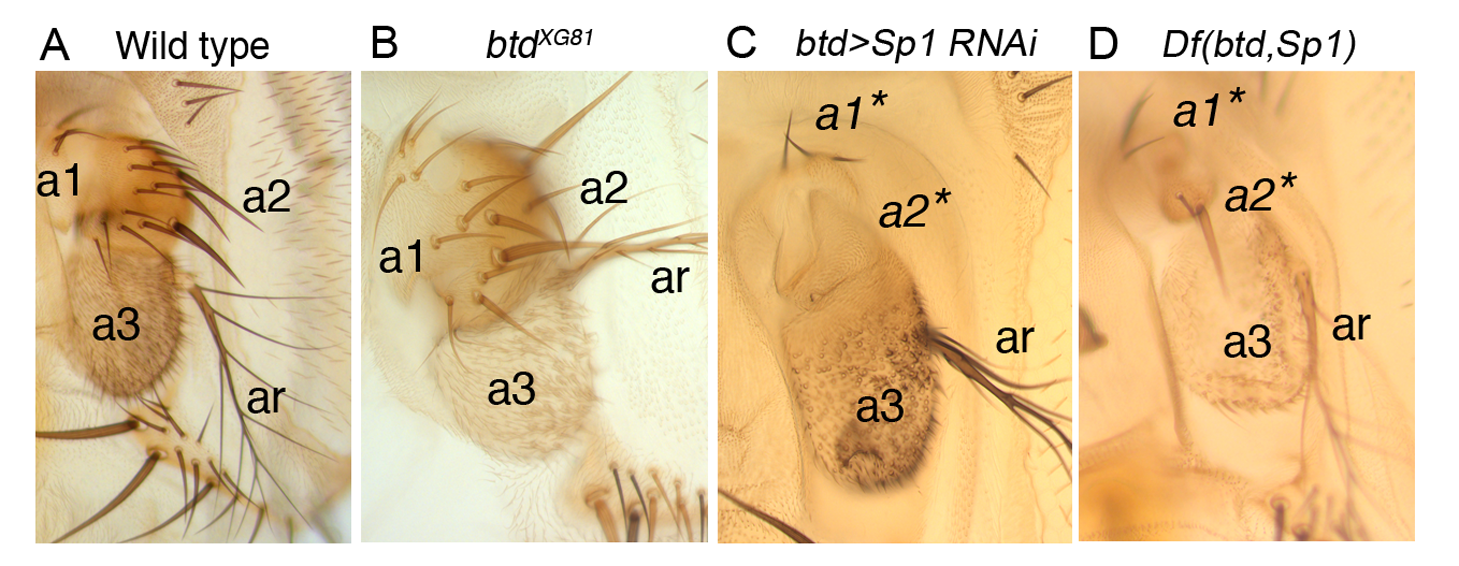

Supplement: Figure S5 — Sp1, but not btd, is required for antennal growth. All antennae are labeled with: 1st segment (a1), 2nd segment (a2), 3rd (a3) and arista (ar). (A) Wild type antenna. (B) Completely btdXG81 mutant antenna marked by y of the geneotype: yw btdXG81 FRT19A/ubi-GFP M FRT19A; Dll-Gal4, UAS-flp. No phenotype is observed in the mutant antenna. (C) btd-Gal4; UAS-Sp1i reduces the size of the a1 and a2 antennal segments. Compare to (A). (D) Antenna of the genotype yw Df(btd,Sp1) FRT19A/ubi-GFP M FRT19A; Dll-Gal4, UAS-flp where the a1 and a2 segments are greatly reduced. (1.34 MB TIF) [file pgen.1001001.s005.tif]

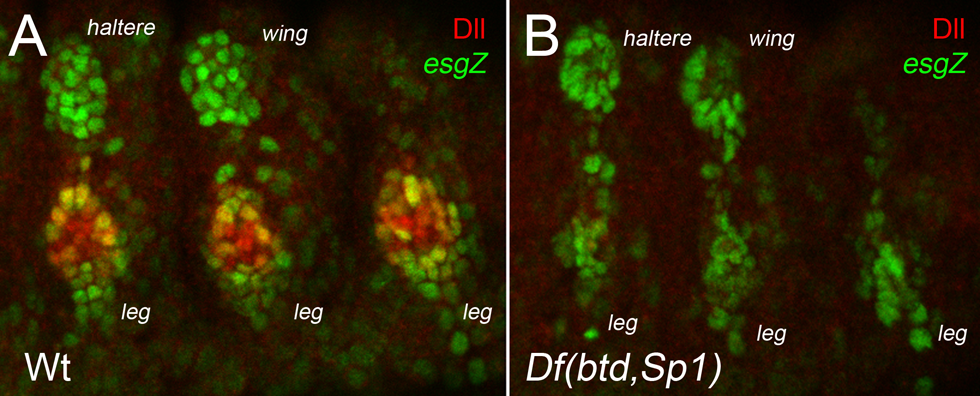

Supplement: Figure S6 — btd and Sp1 mutant embryos fail to maintain Dll expression. Thoracic regions of stage 14 embryos stained for ß-Gal (esg-LacZ, green) and Dll (red). Anterior is left and dorsal is up. (A) Wt embryo showing the thoracic appendage primordia (legs, wing and haltere primordia). (B) Df(btd,Sp1) mutant embryo that fails to maintain Dll expression, compare it to (A). (0.71 MB TIF) [file pgen.1001001.s006.tif]

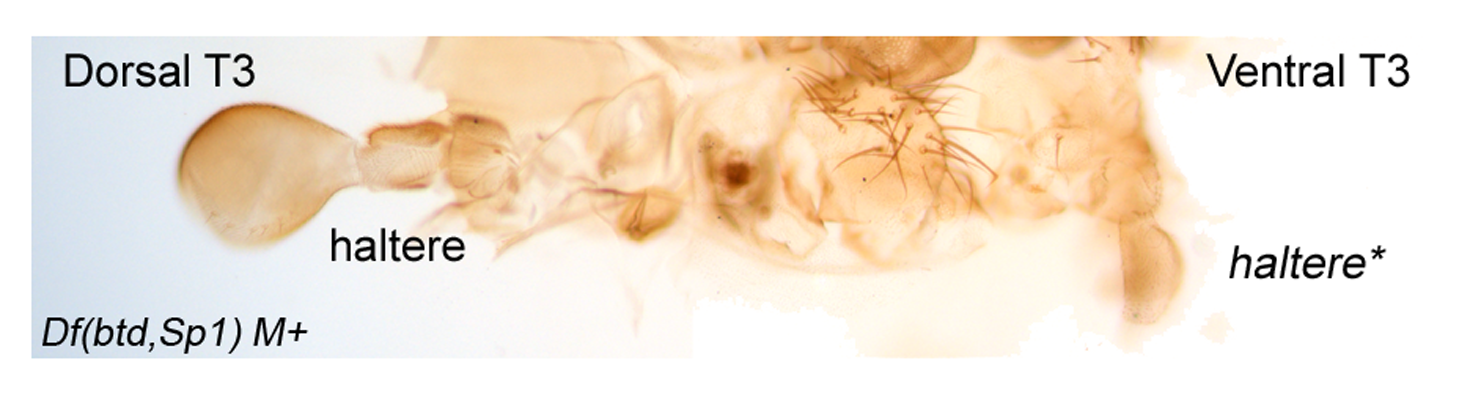

Supplement: Figure S7 — Ventral to dorsal transformation in the absence of btd and Sp1. Hemi-third thoracic segment of a fly of the genotype yw Df(btd,Sp1) FRT19A/ubi-GFP M FRT19A; Dll-Gal4, UAS-flp where the third leg is transformed to an haltere (asterisks). Dorsal is to the left and ventral is to the right. (0.62 MB TIF) [file pgen.1001001.s007.tif]

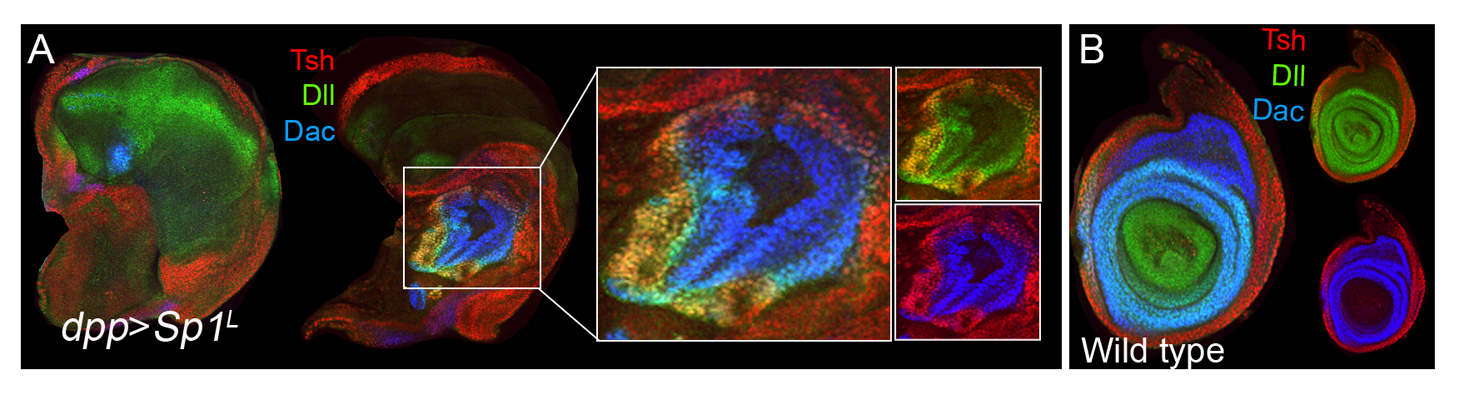

Supplement: Figure S8 — Ectopic expression of Sp1 induces leg development in the wing disc. (A) dpp-Gal4; UAS-Sp1L induces the ectopic expression of the leg PD genes Dll (green), dac (blue), and tsh (red) in the wing imaginal disc. Two planes of focus are shown. Note that the tissue where Dll, dac and tsh are ectopically induced (white square) is organized as a wild type leg imaginal disc. (B) A wild type leg imaginal disc shown for comparison. (0.90 MB TIF) [file pgen.1001001.s008.tif]

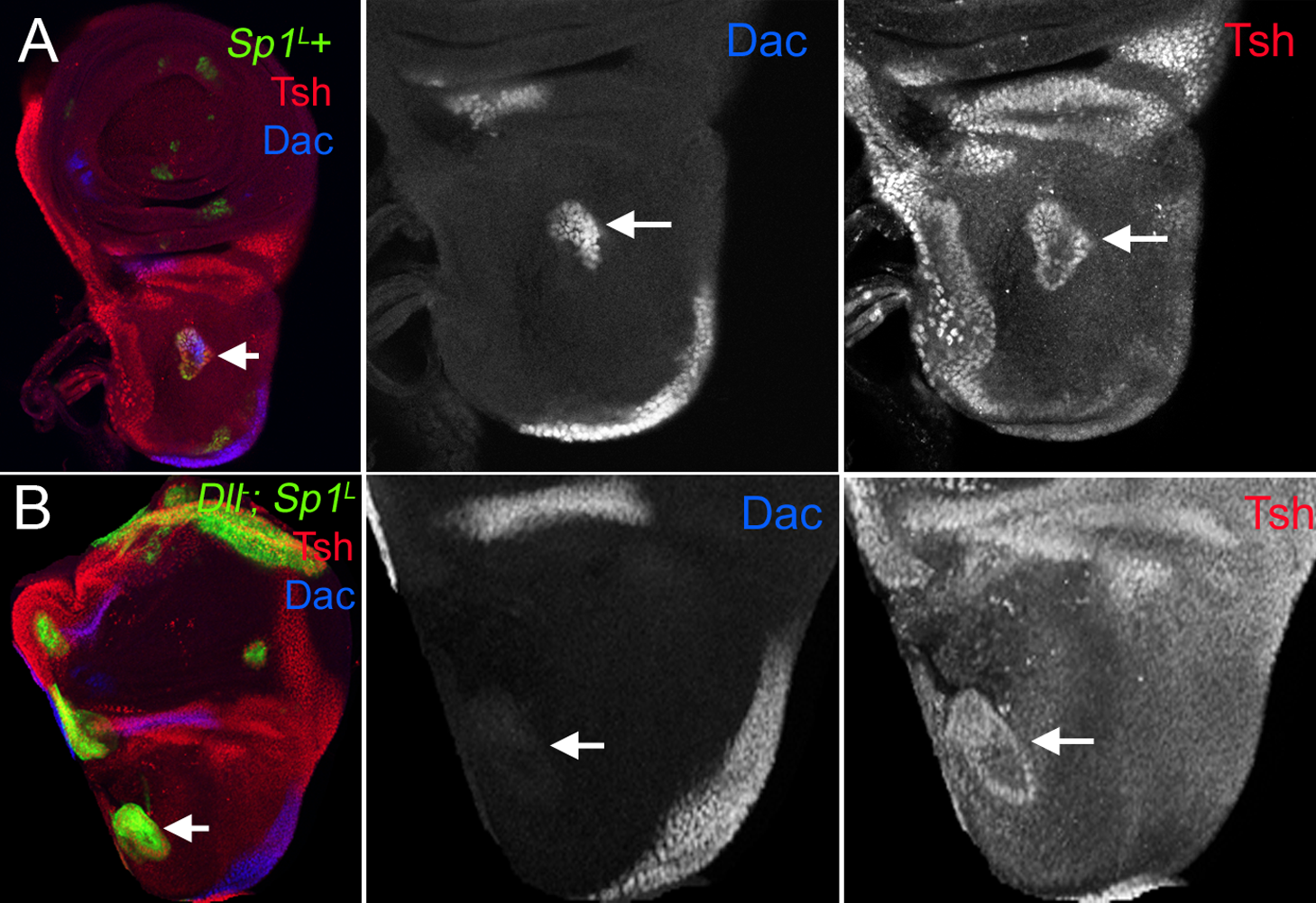

Supplement: Figure S9 — Sp1 requires Dll to induce leg development. (A) Sp1L ectopic expression clones in the wing disc induce the expression of the leg genes dac and tsh (arrows). Clones are generated 48-72 hrs AEL. Note that Sp1 is better able to induce dac expression in the notum that in the wing pouch. (B) Dll-; Sp1L+ MARCM clones fail to induce dac expression (arrows). However, these clones retain the ability to activate tsh. Clones are generated 48-72 hrs AEL. (1.76 MB TIF) [file pgen.1001001.s009.tif]

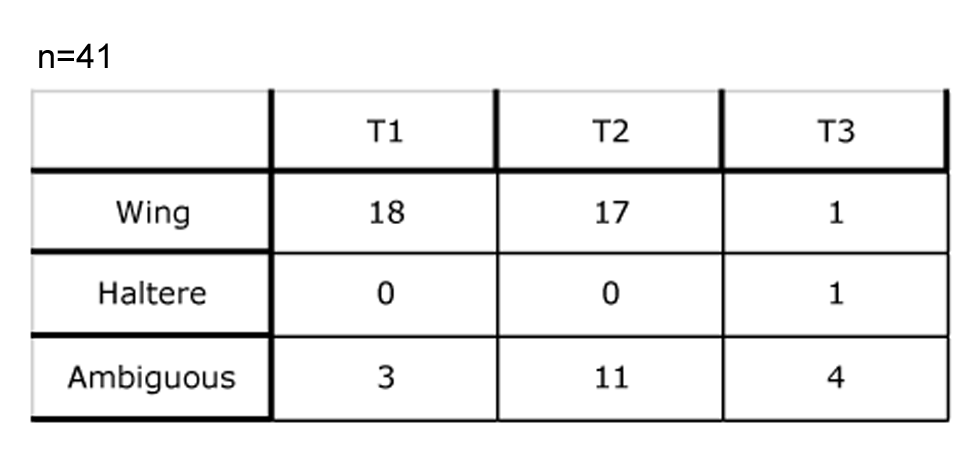

Supplement: Table S1 — Summary of ventral to dorsal transformations. We scored the number of animals of the genotype yw Df(btd,Sp1) FRT19A/ubi-GFP M FRT19A; Dll-Gal4, UAS-flp that had a leg transformation to a dorsal appendage (wing or haltere) in any of the three thoracic segments. The ambiguous category includes those animals that had dorsal transformations but could not be unambiguously scored as wing-like or haltere-like. Total number of animal counted = 41. (0.08 MB TIF) [file pgen.1001001.s010.tif]
